# Learning stable and predictive network-based patterns of schizophrenia and its clinical symptoms

**DOI:** 10.1038/s41537-017-0022-8

**Published:** 2017-05-16

**Authors:** Mina Gheiratmand, Irina Rish, Guillermo A. Cecchi, Matthew R. G. Brown, Russell Greiner, Pablo I. Polosecki, Pouya Bashivan, Andrew J. Greenshaw, Rajamannar Ramasubbu, Serdar M. Dursun

**Affiliations:** 1 Department of Computing Science, University of Alberta, Edmonton, AB Canada; 2 Alberta Machine Intelligence Institute (AMII), University of Alberta, Edmonton, AB Canada; 3 Department of Psychiatry, University of Alberta, Edmonton, AB Canada; 4 IBM T.J. Watson Research Center, Yorktown Heights, NY 10598 USA; 5 Department of Electrical and Computer Engineering, University of Memphis, Memphis, TN 38152 USA; 6 Department of Psychiatry, University of Calgary, Calgary, AB Canada

## Abstract

Schizophrenia is often associated with disrupted brain connectivity. However, identifying specific neuroimaging-based patterns pathognomonic for schizophrenia and related symptom severity remains a challenging open problem requiring large-scale data-driven analyses emphasizing not only statistical significance but also stability across multiple datasets, contexts and cohorts. Accurate prediction on previously unseen subjects, or generalization, is also essential for any useful biomarker of schizophrenia. In order to build a predictive model based on functional network feature patterns, we studied whole-brain fMRI functional networks, both at the voxel level and lower-resolution supervoxel level. Targeting Auditory Oddball task data on the FBIRN fMRI dataset (*n* = 95), we considered node-degree and link-weight network features and evaluated stability and generalization accuracy of statistically significant feature sets in discriminating patients vs. controls. We also applied sparse multivariate regression (elastic net) to whole-brain functional connectivity features, for the first time, to derive stable predictive features for symptom severity. Whole-brain link-weight features achieved 74% accuracy in identifying patients and were more stable than voxel-wise node-degrees. Link-weight features predicted severity of several negative and positive symptom scales, including inattentiveness and bizarre behavior. The most-significant, stable and discriminative functional connectivity changes involved increased correlations between thalamus and primary motor/primary sensory cortex, and between precuneus (BA7) and thalamus, putamen, and Brodmann areas BA9 and BA44. Precuneus, along with BA6 and primary sensory cortex, was also involved in predicting severity of several symptoms. Overall, the proposed multi-step methodology may help identify more reliable multivariate patterns allowing for accurate prediction of schizophrenia and its symptoms severity.

## Neuroimaging: Brain connectivity pattern predicts symptom severity

Brain network analyses from functional magnetic resonance imaging (fMRI) data may help diagnose schizophrenia and predict symptom severity. Detecting neuroimaging patterns requires large-scale analysis across multiple data sets. Mina Gheiratmand and colleagues from the University of Alberta, along with researchers at the IBM T.J. Watson Research Center analyzed brain imaging data from the Function Biomedical Informatics Research Network, a study designed to test the reproducibility of brain scan results taken on different fMRI machines from people with schizophrenia and schizoaffective disorders, as well as healthy controls. They studied brain networks at different levels of resolution from data gathered while study participants conducted a common auditory test. The researchers showed that they could discriminate between patients with schizophrenia and controls with 74% accuracy across multiple neuroimaging sites using the strength of connection in a functional network. They observed the most robust and discriminative connectivity differences between the thalamus and primary motor and sensory cortices as well as between the precuneus and other brain regions. Moreover, they could determine symptom severity based on the connectivity changes involving these areas. This new approach towards finding objective, reliable neuroimaging biomarkers for schizophrenia and its severity could be used for diagnosis and to assess disease progression and therapeutic efficacy.

## Introduction

An ultimate question in functional magnetic resonance imaging (fMRI) studies of schizophrenia is whether one can identify combinations of statistical features extracted from the data that can serve as reliable statistical (bio)markers of the disease, capable of accurately discriminating between schizophrenic patients and controls, which are reproducible (stable) across multiple datasets. Here, we define biomarkers as multivariate patterns, i.e., specific combinations of sets of features, rather than individual features. A traditional approach to finding biomarkers is to perform mass-univariate analysis, i.e., to compare empirical distributions of each feature, independently of the other features, across the two groups of subjects, e.g., patients vs. controls, to test whether the distributions of the feature in those populations are different. However, statistical significance of individual features may neither be a necessary nor a sufficient criterion for discriminating between the two groups, and must be augmented with a wider set of requirements, including (but not limited to) generalization accuracy and stability, as discussed later in this paper. In addition, we propose that the ability to accurately predict symptom severity based on neuroimaging features has an equal, if not greater, importance than the binary disease classification, since it can lead to a more objective measurement-driven characterization of schizophrenia. Emphasizing the importance of objective measurements in psychiatry is one of the central ideas of the recently proposed Research Domain Criteria (RDoC)^1^ initiative of NIMH.

While univariate hypothesis testing simply reveals the cross-group differences between the empirical distributions of *each individual feature*, *on a fixed dataset*, multivariate discriminative models, i.e., classifiers built using *feature (sub)sets*, attempt to predict whether *a previously unseen subject* can be diagnosed with schizophrenia or not. Predictive modeling has potential applications in practical settings, for example, early diagnosis of schizophrenia based on neuroimaging data. Aiming to better understand abnormalities associated with the disease, we focus on interpretable predictive models as opposed to black-box classifiers; particularly, we use feature subset selection in order to identify features most relevant to predicting the disease and the severity of the symptoms. Note that the discriminative task can be more challenging than significance testing: for example, the use of significant (low *p*-value) features in fMRI data does not always result in accurate classification,^2^ and statistically significant variables are not necessarily the best predicting variables.^3^ Thus, a combination of both statistical significance and classification accuracy criteria provides a more complete characterization of candidate features in terms of disease relevance. Finally, *stability*,* or reproducibility*, of the predictive subsets of features across varying data subsets and across different classification methods is another important criterion that should be included when evaluating potential biomarkers.

We focus on the brain’s functional networks derived from functional MRI data,^4–6^ which is a rich source of features highly relevant to schizophrenia^2, 7, 8^ and other psychiatric disorders.^9, 10^ The association between schizophrenia and abnormal brain connectivity dates back to early work by Wernicke^11^ and Bleuler.^12^ It is often referred to as a “dysconnection” hypothesis,^13, 14^ where “dysconnection” accounts for a range of network dysfunctions, beyond simply the “disconnection”, or reduced connectivity. Beyond continued exploration of abnormalities in *anatomical* networks,^15–17^ numerous recent studies^15, 18^ including this work focus instead on disrupted *functional* connectivity, which may have greater potential for capturing the dynamic system properties of a brain “in action”.

We performed fully data-driven, brain-wide analysis of functional networks, defined as thresholded correlation matrices across voxel time-series,^19^ and explored several types of graph features, such as link-weights (correlations) and voxel degrees. In addition to the voxel-level networks, we also evaluated functional networks at a coarser level using supervoxels (defined as 4 × 4 × 3 clusters of adjacent voxels). Such subsampling permitted more comprehensive whole-brain link analysis due to a considerable reduction of dimensionality. Previous work^2, 18, 20^ has demonstrated that functional network features are highly informative when discriminating between schizophrenic patients and controls using multivariate predictive approaches. Our objective here is to investigate the extent to which such findings generalize to different datasets involving different groups of patients and experimental paradigms (i.e., the Auditory Oddball task).

We used the FBIRN multi-site dataset, where cross-site variability introduces an additional challenge. Unlike most recent large-scale functional-connectivity studies of schizophrenia that involve resting-state fMRI,^18, 21^ we focused on a task-based paradigm (Auditory Oddball) which may reveal different aspects of anomalous functional networks, compared to those identified by resting-state studies. We evaluated discriminative ability, in addition to statistical significance and stability, of several types of functional network features using several state-of-art classifiers and the leave-one-subject-out cross-validation (CV) setup.

Overall, link-weights (that is, correlations) in a supervoxel-level functional network were the most discriminative features, achieving 74.0% classification accuracy compared to 51.6% chance level; their performance was followed by node-degrees (70% accuracy). Our learning systems used data from many sites, which is a more challenging task than learning from a single site.^22^ This increased challenge is due to the larger variability in the FBIRN multisite dataset introduced by the differences in the image acquisition equipments across sites (see supplementary Table S1 for scanner details per site) as well as higher patient sample heterogeneity, compared to previous single-site studies that used a homogeneous patient group.^2^ As such, our prediction accuracy is quite encouraging, matching or exceeding the results of similar multisite studies.^18, 21, 23^ (Also, see the Discussion section where we outline the possibility of an overly optimistic classification accuracy results reported elsewhere.)

We also evaluated these features in the context of predicting symptom severity, thus relating functional network disruptions to behavioral metrics. We explored the ability of link-weight features in predicting symptoms severity, indicated by Scales for Assessment of Negative Symptoms (SANS)^24^ and Scales for Assessment of Positive Symptoms (SAPS).^25^ Specifically, we used a sparse (i.e., variable-selection-based) multivariate regression approach known as elastic net (EN) to generate interpretable regression models (corresponding to 9 SANS and SAPS Global Rating Scales) and evaluated them using leave-one-subject-out CV, similarly to the classification models discussed above. The predicted symptom severity scores resulting from this approach were significantly correlated with actual scores (Spearman *ρ* between 0.2 to 0.5), for the following five Global Rating Scales: inattentiveness, bizarre behavior, positive formal thought disorder, avolition/apathy and alogia. These models also allowed us to identify stable predictive subsets of link-weight features, which were selected by the EN model across all CV data subsets. Note that most prior work considers primarily univariate correlations between symptom scales and features of interest^18^; we believe, this work represents the first to actually predict schizophrenia scales via multivariate regression approach using the whole-brain functional connectivity features.

The most statistically significant, discriminative and stable connectivity disruptions in schizophrenia observed in this study involved increased correlations between thalamus and primary motor/primary sensor cortex, as well as between precuneus (BA7) and thalamus, putamen, and areas BA9 and BA44; precuneus was also implicated in abnormally high network degrees. The precuneus was also highly involved in the stable, most-predictive links selected by sparse multivariate models for prediction of clinical symptom severity. BA6 (premotor cortex) and primary sensory cortex were two other areas that played an important role in predicting, with statistically significant accuracy, all five symptom severity scales: inattentiveness, bizarre behavior, positive formal thought disorder, avolition/apathy and alogia. It is of interest that, while several brain areas, and especially precuneus, were consistently involved in both disease classification and scale prediction models, the specific links found by sparse regression to be most predictive about the scales were not necessarily among the most-discriminative links, and vice versa.

The contributions of this paper are as follows:

1. We demonstrate that combinations of functional network features (specifically, supervoxel-level link-weights) can accurately predict not only the presence of schizophrenia, but also the severity of its clinical symptoms. We observe that the most significant functional network disruptions involve abnormal increase of thalamo-motor cortex correlations, confirming prior resting-state observations on this task-based (Auditory Oddball) data. Furthermore, precuneus is consistently involved in multiple network links with abnormally increased correlations.
2. We observe considerable “hyperconnectivity” in patients, i.e. higher than normal weights (correlations) along all significant links, as well as increased node-degrees at all significant nodes. Our observations are consistent with the findings of Yang et al.^26^ who report network hyperconnectivity (especially in the fronto-parietal network) in schizophrenia, resulting from an increased excitation to inhibition ratio simulated using a neural model.
3. From the methodological point of view, we propose a stricter evaluation framework for candidate neuroimaging-based “statistical biomarkers” of schizophrenia, including cross-dataset stability and generalization accuracy. We argue that such an approach can result in more reliable markers as compared to those produced by univariate statistical tests alone.

## Results

### Mass-univariate statistical hypothesis testing

A large proportion of both ss-log-degree and ss-link-weight features (see Methods for a description of the features) were significantly different across the two groups: more than 10,000 ss-log-degree features out of 26,949 and more than 100,000 ss-link-weight features out of 161,596 survived the false discovery rate (FDR) correction for multiple comparisons; moreover, around 700 ss-log-degree features and around 14,500 ss-link-weight features also survived the Bonferroni test. The lowest *p*-value attained by ss-log-degree and ss-link-weight features were around 10^−12^ and 10^−19^, respectively (see Supplementary Fig. S2 in the Supplemental Material for more details).

### Stability analysis

However, high statistical significance does not yet guarantee feature stability across varying datasets. As described in the Methods section, for the discriminative task, we generated 95 different subsets of our data, where each subset was obtained by leaving out one subject, i.e. all 4 runs associated with that subject. Next, we performed the two-sample *t*-test on each of the data subsets, (called CV-folds). Finally, we computed the intersection of all Bonferroni-surviving feature subsets, across the CV-folds. Only 426 ss-log-degree features survived this “stable Bonferroni” test, as compared to 700 features surviving Bonferroni on the single full dataset.

**Fig. 1:**
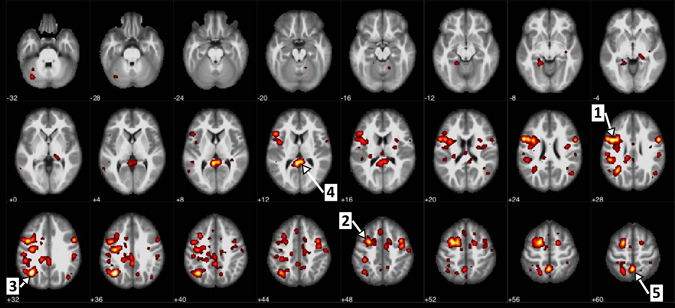
A stable subset of statistically significant (Bonferroni-surviving) site-standardized (ss) log-degree features, across 95 data subsets corresponding to leave-subject-out CV. For each data subset, a two-sample *t*-test was performed to select the subset of features surviving Bonferroni correction. The intersection of all such subsets contains 426 “stable Bonferroni” voxels (vs. 700 that survived Bonferroni on the whole dataset but were not necessarily stable). Note that mean values of the site-standardized log-degree, as well as the corresponding log-degree features, at all highlighted voxels, were higher in schizophrenic group than in the control group (see Supplementary Fig. S3). The *numbered arrows* in the figure point to the most-significant (smallest *p*-value) largest clusters, which included: (1) left BA6 (precentral gyrus, inferior frontal gyrus); (2) left BA6 (middle frontal gyrus); (3) left BA39 (lateral occipital cortex, superior division); (4) left BA30 (cingulate gyrus, posterior division); and (5) left BA7 (precuneus). Also, see Supplementary Table S5 for the MNI coordinates of the clusters’ local maxima). For visualization purposes, the original-resolution statistical maps are upsampled (to 2 × 2 × 2 × mm), thresholded based on intensity and cluster size, and smoothed using a Gaussian kernel (5 mm FWHH) in bspmview.^28^ The original statistical *p*-map is provided in supplementary information below Supplementary Table S5

The regions corresponding to stable Bonferroni-surviving ss-log-degree features are shown in Fig. 1. Brain areas that contained the most-significant voxels (in *yellow*) involved Brodmann areas BA39.L (left), BA7.L, BA6.L and BA30.L (see Lacadie et al.^27^ for details of Montreal Neurological Institute (MNI) space to Brodmann area conversion). More specifically, the cortical brain areas corresponding to the largest clusters with the lowest *p*-values included lateral occipital cortex, precuneus cortex, precentral gyrus, middle temporal gyrus, and cingulate gyrus, according to the Harvard-Oxford Structural Atlas. (Supplementary Table S5 summarizes details of the eight largest clusters.) It is interesting to note that, for all stable Bonferroni-surviving ss-log-degree features (for all voxels shown in Fig. 1), the corresponding mean ss-log-degree, as well as just mean log-degree (and mean degree) values were higher for the schizophrenic group as compared to controls (also, see Discussion and Supplementary Fig. S3).

As described above, for the other feature type, the ss-link-weights, the number of Bonferroni surviving features was very large (above 14,500), and even the stable subset of those over CV-folds resulted in too many links (10,112 links). Thus, we do not display the stable Bonferroni ss-link-weight features here, since the corresponding plot would be too complex to be informative. Later in the paper, however, we describe application of an even stricter feature subset selection criterion based on predictive accuracy of a multivariate classification model, and present an illustration of a stable (over all CV-folds) subset of best-predictive links.

**Fig. 2:**
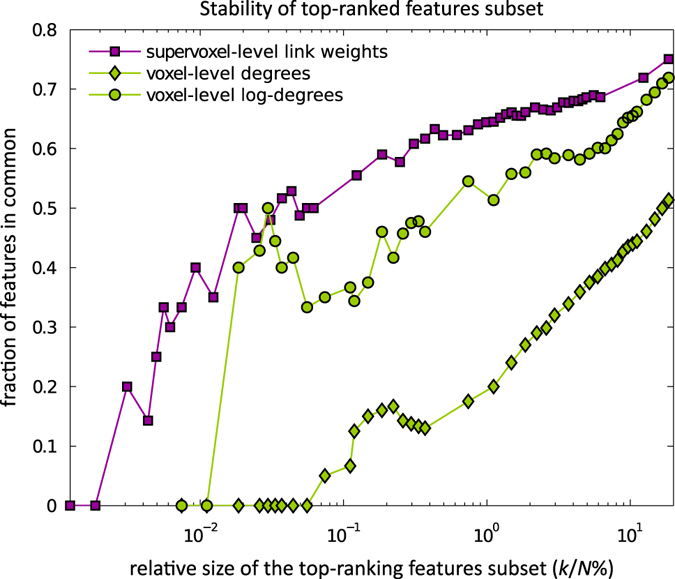
Stability of feature subset selection over CV-folds. Stability is measured as the fraction of features in common among the subsets of *k* top features selected in all CV-folds. Plots present the stability of the non-standardized version of the features: degrees, log-degrees, and link-weights described in Methods. The stability rates for the standardized version of log-degree and link-weight features, ss-log-degree and ss-link-weights, were similar to the non-standardized versions presented here and are not displayed

Next, we evaluated how feature stability varies with the size of feature subsets. For each feature type, and for varying feature subset size *k*, we selected the top *k* most-significant (lowest *p*-value) features on each of the leave-subject-out data subsets (CV-folds) and computed the ratio of the features common to all CV-folds. Figure 2 shows this fraction (i.e., the number of common features divided by *k*) for link-weight and log-degree features, as well as for just degree features, plotted against the (normalized) feature subset size (log-scale) measured by percent *k/N*, where *N* is the total number of features of a particular type (we use the normalization since *N* can be different for different feature types). Obviously, the general trend is that the ratio of common features increases with *k*, and for *k* = *N* (i.e., all variables used), the overlap between features selected in different folds is 100% (each fold uses all the features). Thus, here, we only focus on the first part of the plot, which includes only up to about 20% of the total number of features (*N*), where every 1% increase in *k* is equal, respectively, to about 1616 and 270 features for the link-weights and log-degrees (and degrees).

Our main observation here is that the link-weight feature type appears to be the most stable, followed by the log-degree feature; note that both features are much more stable than the original degrees, which is another reason (besides better classification accuracy) for choosing to focus on them here. The stability curves for the site-standardized link-weights and log-degrees were similar to their non-standardized versions and are not shown here.

### Learning classifiers to test discriminative ability of different feature types

**Fig. 3:**
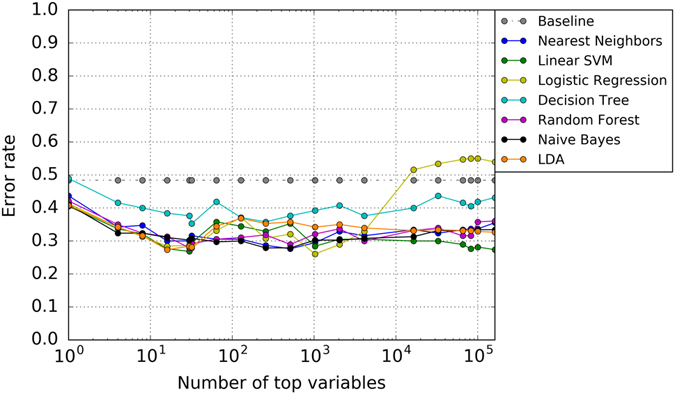
Classification of patients vs. controls based on ss-link-weight features. Y-axis shows the average CV error over 95 leave-subject-out folds (see the Supplementary Fig. S4 for the corresponding FP and FN rates) for different classifiers, and for varying size of top-ranking feature subset shown on *x*-axis

Figure 3 shows average classification error (*y*-axis), over 95 CV-folds, obtained on test data (leave-subject-out data subset) by various classifiers trained on the remaining training data, for the ss-link-weight feature, and for different feature subset sizes shown on the *x*-axis for patients vs. controls. Recall that each subset consists of *k* lowest *p*-value features selected separately for each CV-fold, to avoid “double-dipping” during the feature-selection process. Note that for every *k*, all *k* top-ranking features in that CV data subset are used as features in the model, not just the CV stable subset. We will later analyze the stable features for the winning *k*-size subset, i.e., the subset that yielded the lowest classification error. The classification error based on the ss-log-degree features is given in Supplementary Fig. S4.

The most accurate classification, on average, was achieved using the ss-link-weight features: 26.8% error (73.2% accuracy) using linear support vector machines (SVM) on subsets of only 30 features out of a total of 161,596 at each CV-fold, and 26.0% (74.0% accuracy) using logistic regression with 1024 features. (The lowest error rate and feature number for all other classifiers is summarized in the Supplementary Table S6.) For more details, including the false positive (FP) and false negative (FN) errors, see Supplementary Fig. S4 in the Supplemental Material. The ss-log-degrees yield somewhat less accurate prediction, 30% error at best, using more than 10,000 features (Supplementary Fig. S4). Note that classification performance is quite consistent among majority of the classifiers, at least for the smaller values of *k*. The cross-classifier stability of the results suggests that corresponding subsets of top *k* features may indeed contain discriminative information that is reliably picked up by many classifiers, as opposed to a potential fluctuation in the error estimate of a particular classifier. Note that the reported error is the lowest average error achieved by each model.

**Fig. 4:**
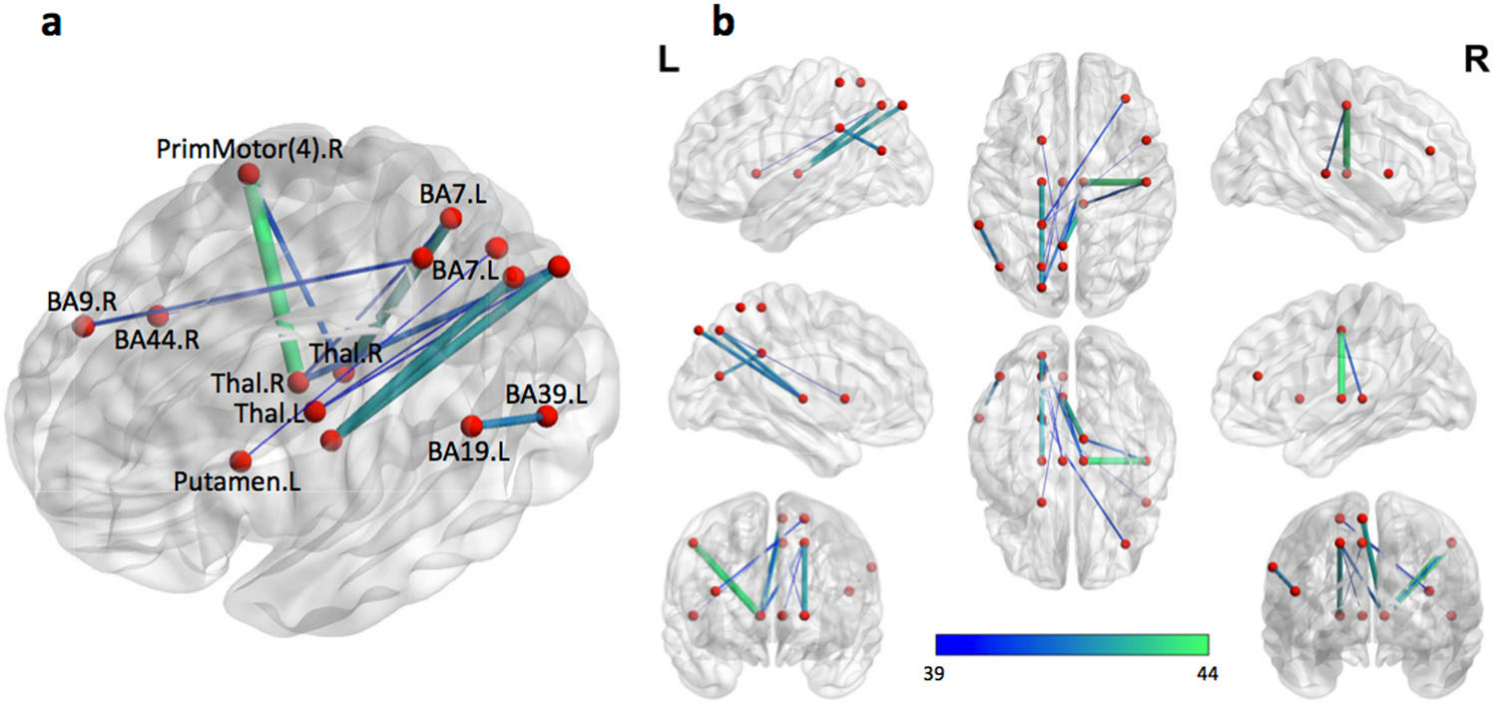
Map of 13 stable (supervoxel-level) links common to all subsets of 30 top-ranked (lowest *p*-value) links, over 95 different CV-folds (leave-one-subject out data subsets). **a** Custom view. **b** Full view of the links presented in **a**. The *color bar* shows –ln(*p*-value). Labels show the Brodmann areas (BA) corresponding to the end nodes. *X*, *Y*, *Z* coordinates of the nodes in the MNI space are given in Supplementary Table S7. A summary of links is presented in Supplementary Table S9 (see also Supplementary Fig. S9 for a reference map of all BAs)

In summary, it appears that the site-standardized link-weights were not only the most stable, but also most accurate features for discriminating between schizophrenia and control in our study. Figure 4a, b shows a subset of the 13 most stable and predictive links, i.e., the links common to all top-30 ranked link subsets corresponding to the most-predictive ss-link-weight features, over all CV subsets (recall that, as shown in Fig. 3, linear SVM achieved lowest error at *k* = 30 top features). Note that all of the above 13 links were also top-ranking based on the t-tests ran on all of the data. A complete list of the links are presented in Supplementary Table S9, with a list of nodes (supervoxels) *X*, *Y, Z* coordinates in the MNI space, functional (BA)^27^ and anatomical labels presented in Supplementary Table S7.

We observed that the most significant links, i.e., the ones with the lowest *p*-value on the full dataset, connect the thalamus to primary motor (next to the primary sensory) cortex. Next, we observed multiple disrupted connections involving precuneus (BA7), including highly significant links connecting it to thalamus, as well as to right Brodmann areas BA9, BA44 (inferior prefrontal gyrus) and to the putamen. Note that the left BA7 and BA39 areas were already implicated in abnormal ss-log-degree features in Fig. 1.

Furthermore, we observe, for all 13 stable predictive links, and also for all Bonferroni-surviving links, that the corresponding mean link-weights were higher for schizophrenic group as compared to the control group; see Supplementary Fig. S5 in Supplementary Information, which demonstrates that, indeed, a large fraction of whole-brain connections has increased link-weight in schizophrenic patients, even after regressing out motion parameters to rule out the possibility of motion-related artifacts; see the Discussion section.

### Disease symptom severity prediction

Since in the discrimination study, the whole-brain (supervoxel-level) link-weights were the most discriminative features related to the presence of schizophrenia, we tested whether this set of features could also be informative about the disease severity. Note that we are considering the entire set, and are not focusing on just the 13 features that were best for the previous task.

**Fig. 5:**
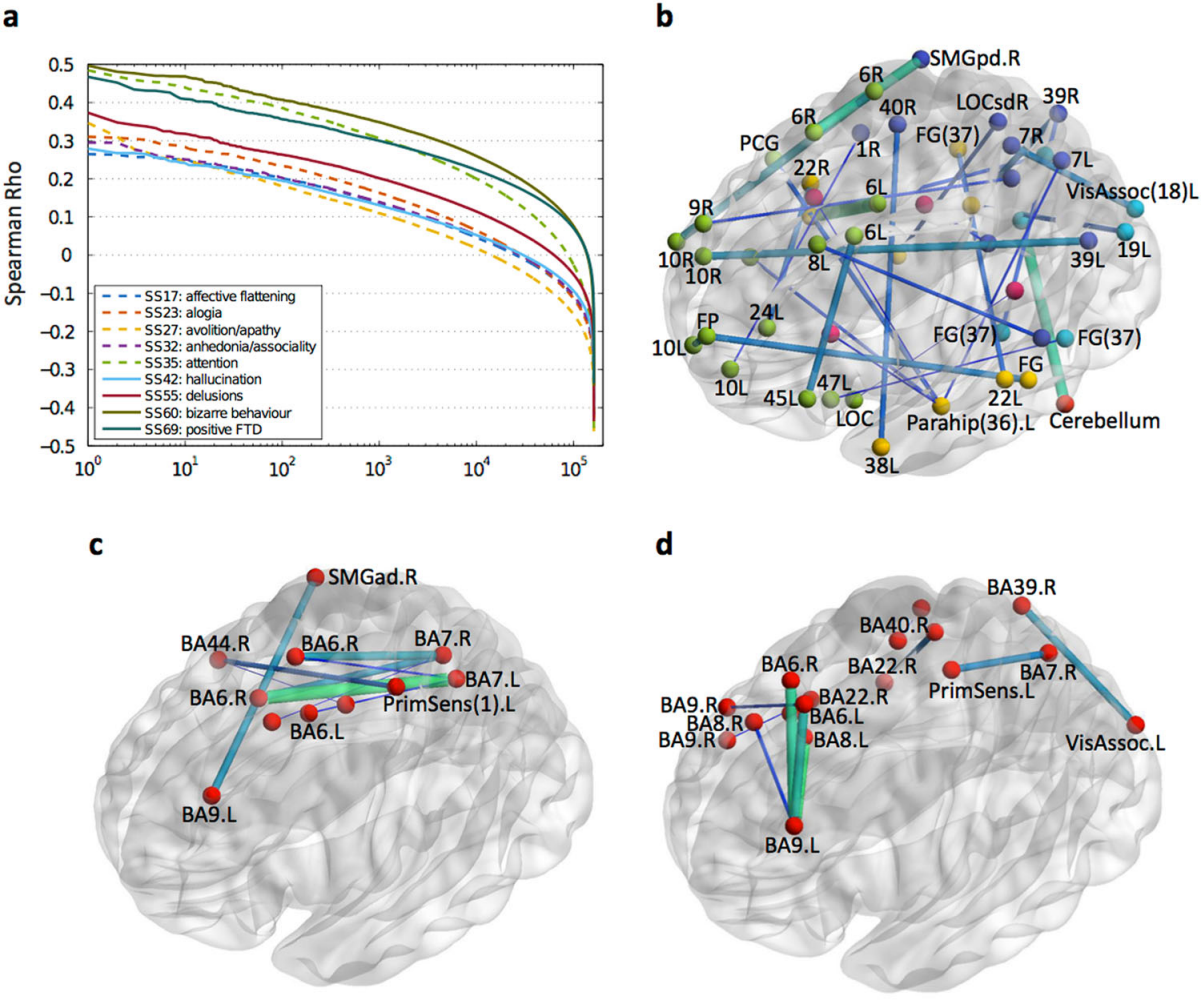
**a** Correlations between all link-weight features and scales. SANS and SAPS scales are shown using *dashed* and *solid lines*, respectively; **b**–**d** stable across CV-folds subset of most-predictive links selected by EN for predicting the global rating of **b** attention, **c** severity of bizarre behavior and **d** positive formal thought disorder. Links thickness and color is scaled based on the average weight of links across CV-folds in the EN model. In **b**, nodes are color-coded based on the lobe they are located in, as follows: frontal (*green*), parietal (*dark blue*), temporal (*yellow*), occipital (*blue*); cerebellum (*red*) and white matter (*magenta*). (see also Supplementary Fig. S9 for a reference map of all BAs; for the scale SS35 [attention], the third best model was used for visualization, as the links map for the best model was very complex [129 out of 700].)

Figure 5a shows, for each of the 9 scales, the Spearman correlations of that scale with each link-weight feature, in descending order. (Pearson correlations yielded similar results.) For all scales, symptom severity varies in the range {0, 1, 2, 3, 4, 5}, with 0 representing no symptom and 5 representing severe symptom. The three scales, Global Rating of Attention, Global Rating of Severity of Bizarre Behavior, and Global Rating of Positive Formal Thought Disorder achieved the highest correlations (of almost 0.5); for the remaining scales, the best correlations were between 0.26 and 0.38.

Next, we evaluated the generalization ability of the link-weight features, i.e., focused on predicting the scales based on sparse multivariate regression model (EN), which includes link-weights as features (predictors). Table 1 shows the best results across all the EN parameter settings discussed in the Methods section (see also Supplementary Information): this required performing 135 tests (9 scales × 5 number of variables × 3 *λ*_2_ values).

**Table 1:** Scale prediction results

For each of the nine Global Rating Scales, the largest Spearman correlation coefficient and the corresponding *p*-value are presented. *Gray rows* correspond to negative symptoms scale (SANS) and white background rows correspond to positive symptoms scale (SAPS). The last two columns show whether the *p*-values of the correlation survived Bonferroni and/or FDR correction. Scales that survived FDR are displayed in bold

The EN model built using link-weight features predicted several SANS and SAPS scales with relatively high accuracy and statistical significance. The resulting *p*-values of Spearman correlations showed that five survived (*p* < 0.05, including the FDR correction to correct for the multiple comparisons to evaluate multiple parameter setting for EN for multiple scales): SS35 (attention), SS60 (severity of bizarre behavior), SS69 (positive formal thought disorder), SS27 (avolition-apathy), and SS23 (alogia). Three of those scales, SS35, SS60, and SS69, also survived Bonferroni correction. These also had the lowest mean absolute error as summarized in Table 1. For the remaining four scales (Global Rating of Affective Flattening, Anhedonia-Asociality, Severity of Hallucination, and Severity of Delusions), the correlation values varied between −0.04 and 0.12 with non-significant *p*-values (0.12–0.87).

For the scales surviving FDR, the best prediction was achieved with the EN model using the following *λ*_1_ parameters (measured by the number of features selected, corresponding to number of non-zero weights): for SS23, 100 features; for SS27, 300 features; for SS35, 700 features; for SS60, 50 features; and for SS69, 50 features; see Supplementary Information for *λ*_2_ parameter for the best models. Supplementary Figure S6 shows the EN model prediction for SS35 for all parameter settings. EN prediction results for all nine scales, as well as the average predictor coefficients and feature selection stability are shown in three separate figures in the Supplementary Information Appendix.

Finally, we considered the most-predictive features selected by the EN model, which, as a sparse method, has an inherent variable-selection property. Figure 5b shows, for scale SS35 (attention), the stable subset of 28 links (out of 300 links) selected in all CV leave-one-subject-out subsets. The full view is presented in Supplementary Fig. S7; list of all connections is presented in Supplementary Table S10a. We demonstrated the stable links for the third best model, which achieved comparable prediction to the best model (Spearman’s rho = 0.4670, *p*-value = 2.34 × 10^−11^ vs. rho = 0.4894, *p*-value = 1.79 × 10^−12^), but used a much lower number of features (300 vs. 700). For the sake of interpretability of the visualized link maps, our algorithm picks the model with the smallest number of features that has a significant correlation within 5% of the highest correlation; see also Supplementary Fig. S6 for EN model performance for all parameter settings. The stable links map for the best model (700 nonzero features in the EN model) included 129 links and was too complex to display here; the map is presented in Supplementary Fig. S7. In total, 48 nodes were involved, including 17 frontal, 11 temporal, 11 parietal, 6 occipital, 4 white matter, and 1 cerebellum. We observed numerous long-range connections. The strongest connections (i.e., the features with the largest average coefficient in the EN model), displayed in *bright green* and *thicker links* in the Figure, consisted in connections from BA6.L to BA22.R (right) and from cerebellum.L to BA19.R. In addition, several links involved parahippocampal cortex (BA36.L) connecting to other areas including BA7.L, BA22 and Precentral Gyrus (both in the right hemisphere).

Figure 5c shows the 9 stable links, out of the 50 links for prediction of the scale SS60 (severity of bizarre behavior); recall that the EN model with 50 nonzero coefficients corresponded to the best average prediction. The full view is presented in Supplementary Fig. S7. Brain areas involved in the predictive links, such as bilateral BA6 and BA7, are quite consistent with the areas involved in significantly disrupted log-degrees (Fig. 1: BA6.L and BA7.L). BA44.R and BA7.L in Fig. 5c are also involved in stable subset of most-discriminative links shown earlier in Fig. 4.

Figure 5d shows the subset of 11 stable links (out of 50 links) for prediction of scale SS69 (positive formal thought disorder). Several connections involved BA9.L, which spanned mainly to areas BA6 and BA8 on the right and left hemispheres, with the strongest links consisting of BA9.L-BA8.L and BA9.L-BA6.R. Several other areas were also involved including BA7, primary sensory cortex (BA1), BA22, BA39, and BA40.

Maps of the stable predictive links for the two remaining scales that survived FDR correction, SS23 and SS27, are given in the Supplementary Fig. S7d, e. A description of the involved links and areas are presented in Supplementary Information.

For each scale, the *X*, *Y*, *Z* coordinates of the end nodes for the stable links are listed in the Supplementary Table S8a–e. We observed consistencies between areas involved in prediction of various scales. For example, areas BA7 (precuneus), BA6 (premotor cortex), and the primary sensory cortex appeared in all five FDR-surviving scales; see also Supplementary Fig. S9. However, overall, patterns of the stable subset of links contributing to different scales’ prediction looked distinct, as can be seen in the visualizations in Fig. 5b–d and Supplementary Fig. S7a–e. Summary of the stable links involved in prediction of different scales is presented in Supplementary Table S10a–e.

Curiously, the most predictive links selected by EN for scales prediction, even including those in stable subsets, were not among the most significantly different (lowest *p*-value) links between the patient and control groups computed on the full dataset. For example, Supplementary Fig. S8 illustrates this for scale SS69. The FDR plot (Supplementary Fig. S8a) shows that the *p*-values of the stable EN-selected features (link-weights), marked in *red*, did not necessarily survive the Bonferroni—or even FDR-corrected thresholds, and the scatter plot in Supplementary Fig. S8b shows that the mean values of the stable EN-selected features were not very different among the patient vs. healthy control group, shown in *y* and *x*-axes, respectively. These observations suggest that the features (link-weights) discriminating patients from controls might be different from those that predict symptom severity. For example, thalamo-cortical connections were heavily involved in classification but were not among the link subsets that were relevant in scale prediction. The feature selection methods in classification vs. regression were different, however, and it would be interesting to use a similar feature selection approach in both tasks before comparing the links involved in classification vs. scale prediction (possible alternative approaches are discussed in the Discussion).

## Discussion

### Methodological messages

We are proposing a multi-step methodology that combines statistical testing, stability, and generalization accuracy in a predictive setting, in order to identify interpretable and reproducible sets of functional connectivity features that can distinguish between patients with schizophrenia and healthy control subjects. We considered two main functional network feature types extracted from the whole brain in response to an Auditory Oddball task (namely, voxel-level degrees and supervoxel-level link-weights) and subjected them to a series of tests, including univariate statistical testing, features stability analysis, and classification performance. While many features (of both feature types) showed statistically significant group-level differences, multivariate subsets of supervoxel link-weights were superior in terms of stability and classification performance. We achieved 74.0% accuracy, vs. 51.6% chance level, in discriminating between patients with schizophrenia and control subjects. This is a promising result as our study involved data from five different sites, meaning we had to overcome higher variability in the data due to differences in image acquisition equipments across different sites (dubbed as the “batch effects”), as well as heterogeneity in the patient group, compared to single-site studies.^2^

We were also able to predict symptom severity related to several measures on the SANS and SAPS Global Rating Scales (these include: attention, bizarre behavior, positive formal thought disorder, avolition/apathy, and alogia), with considerable cross-validated accuracy obtained by applying the EN sparse multivariate regression model to the supervoxel link-weight features from the whole brain: statistically significant, surviving Bonferroni correction, Spearman correlations ranged between 0.2 and 0.5. It is notable that most prior work has considered only univariate correlations between symptom scales and features of interest.^18^

Machine learning approaches have been used in other multi-site schizophrenia datasets, such as MCICshare,^23^ reporting classification accuracies around 70%, with some variations.^29^ Recently, Cheng et al.^18^ reported an overall classification accuracy of 75.8% on a large multicenter dataset that included 398 schizophrenia patients and 375 healthy controls. However, the Cheng et al.^18^ reported accuracy might be an overly optimistic estimate of true generalization accuracy, since the features used in classification were selected based on statistical significance testing on the full dataset, rather than within each CV data subset (fold) as implemented in our paper. That approach is considered “double-dipping” and may typically result in overly optimistic estimates of the generalization error (see Methods). Our prior work^2^ gave an example showing that such double-dipping (i.e., selecting the relevant-to-class-label features on all of the data, including train and test sets), could result in an apparently high accuracy of 93% using Gaussian Naïve Bayes (GNB) on 100 top-ranked degree features, while doing the variable selection properly, i.e. separately on each CV training subset, results into a more appropriate measure of accuracy, which was 20% lower (see also Molla et al.^30^ and van ‘t Veer et al.^31^ for an example based on analysis of genomics data with respect to breast cancer prognosis). Classification accuracies in smaller datasets with a more homogeneous patient group, recruited at a single site, are generally higher^2, 32^ (see also Wolfers et al.^29^ for a review). This highlights the challenges of generalizing to larger datasets. Overall, aiming at achieving high classification accuracy in large datasets is essential in moving towards deployment of neuroimaging and machine learning in clinical applications and thus remains an important direction for future research.

### A closer look at the most-discriminative stable subset of links

We observed that a subset of only 30 top-ranking supervoxel link-weights out of a total of 161,596 provided the lowest CV error (26.8%) using linear SVM. Note that this is the lowest average error on the full dataset involved in leave-one-subject-out CV. Though a slightly lower error of 26.0% was obtained with over 1000 features using the logistic regression classifier, for the purposes of better interpretability and visualization of the results, we focus on the subset of only 30 features, since the corresponding error was comparable to (within 5% from) the lowest error, but achieved on a much smaller feature subset. We see that the most significant link connected thalamus to the primary motor cortex (next to the primary sensory area). Disrupted thalamo-motor/sensory connectivity was previously reported in some studies^18, 33, 34^; however, this prior observation involved only resting-state fMRI data, so it is quite interesting to see similar disruptions in a task-based fMRI setting. The subset also included several disrupted connections from the left superior parietal lobule (precuneus or Brodmann area BA7.L as shown in Fig. 4) to thalamus, as well as Brodmann areas BA9, and BA44, both in the right hemisphere (inferior prefrontal gyrus) and putamen. Abnormal connectivity involving precuneus (BA7) was previously reported in another schizophrenia study based on an auditory language-based task^2^, and also in some other related studies in schizophrenia (e.g., Mashal et al.^35^ with respect to metaphor comprehension). Disruptions in BA44 area (inferior frontal gyrus) has also been reported repeatedly in the literature^36^ on schizophrenia.

### Most-discriminative vs. most-significant brain regions

There was some overlap in brain areas involved in the most discriminative pairwise correlations (link-weights between supervoxels) (Fig. 4) and areas corresponding to the most significant difference in voxels log-degrees (Fig. 1). These included Brodmann areas BA7 (precuneus) and BA39 (lateral occipital cortex) left. Also, the right thalamus, which was heavily involved in the most discriminative links (via thalamo-cortical connections between the right thalamus and the areas BA7.L [precuneous] and primary motor.R cortex), was also among the areas with aberrant log-degrees; coordinates of the local maximum of the cluster located at the right thalamus: *X* = 17.60, *Y* = −29.75, *Z* = −2; see also the 8th and 9th panels in Fig. 1. Other brain areas containing the most-significant voxels included BA6 left and right (precentral gyrus, inferior frontal gyrus) and BA30 (cingulate cortex), while the areas involved in the most predictive links included primary motor (postcentral gyrus) right and BA44.R. Note that the labels for nodes involved in classification represent the center of the supervoxels, each covering a relatively large area of 13.75 × 13.75 × 15 mm. Supervoxel may, therefore, partially involve other adjacent brain regions.

### Direction of the functional connectivity changes

While there is a general consensus on disruption of functional connectivity (in several brain areas) in schizophrenia, so far there is no consensus on the direction of the alteration, with some studies reporting hyper-connectivity and others hypo-connectivity, as well as a mixture of the two.^16, 37, 38^ Here, we observed that a large fraction of whole-brain connections had increased link-weight as well as nodal degrees in schizophrenic patients (Supplementary Figs. S3 and S5). This phenomenon was preserved after regressing out motion parameters (mean absolute and mean frame-to-frame motion displacement) from the features, in a control experiment, to rule out the possibility of motion-related artifacts (Supplementary Fig. S5), as a persistent source of methodological concern in fMRI data analysis.^39, 40^ In addition, our preprocessing focused particularly on eliminating possible motion artifacts, and, as discussed in the Methods section, included standard FSL motion correction, and an additional step involving tCompCorr denoising. We also applied low-pass temporal filtering to further eliminate possible motion artifacts. (Motion parameters were not included as regressors at any stage of our main analyses, including preprocessing, features post-processing, or prediction.)

Our observation of increased connectivity in schizophrenia is consistent with the Yang et al.^26^ report of hyperconnectivity in schizophrenia as a result of an increased excitation to inhibition ratio, simulated using a neural model. Hyperconnectivity in the resting-state default mode network (DMN) in patients with schizophrenia has been reported elsewhere.^16, 38^ Also, DMN deactivation during cognitive tasks may be deficient in schizophrenia,^41^ which might relate to the overall higher functional connectivity in patients vs. controls, similar to our observations in this task-based fMRI study. In addition, some studies have reported increased thalamo-sensorimotor connectivity in schizophrenia,^18, 33, 34^ although they also reported decreased thalamo-frontal connectivity in this context, which was not observed in our analysis (there were no thalamo-frontal links in the stable links subset). This difference in findings may be related to the task-based nature of our data, i.e. the Auditory Oddball paradigm, which could perhaps contribute to an overall increase in observed connectivity strengths compared to resting-state studies.^18^

### Prediction of specific symptoms

In addition to successful classification, link-weight features, extracted from the whole brain, were predictive of the severity of several symptoms in patients, including global rating of attention, severity of bizarre behavior, positive formal thought disorder, avolition/apathy, and alogia. The relationship between aberrations in brain functional connectivity and clinical symptoms as an independent measure of illness severity is yet unknown. While some recent studies have considered the correlation between symptoms and neuroimaging features,^18, 36, 42, 43^ reporting correlations in a range typically below 0.5 (e.g., Cheng et al.^18^ reported below 0.17 correlations between functional connectivity features involving the thalamus and the PANSS scales), only very few studies have attempted multivariate prediction of scales using neuroimaging features.^44, 45^ (Koch et al.^44^ used leave-subject-out support vector regression to predict the PANSS negative scale and activation pattern in ventral striatum in the context of gain anticipation in schizophrenia. Tognin et al.^45^ reported prediction of symptom progression in individuals at ultra-high risk for psychosis at a 2-year follow-up (correlation = 0.34, *p* = 0.026) using cortical thickness and relevance vector regression. While our manuscript was under review, we learned about a recent paper^46^ that reported accuracies as high as correlation = 0.7 and 0.78, respectively in prediction of MCCB cognitive and PANSS symptomatic scores in schizophrenia using multimodal MRI data (fMRI, DTI and MRI). The authors used several levels of feature selection (including removing features with low variability, regression ReliefF feature selection, spatial clustering, and correlation-based feature selection) that reduced the number of voxel-wise features (fALFF, fractional anisotropy, and gray matter) from thousands to dozens, resulting in a much improved accuracy compared to using less or none feature selection prior to regression.

We found consistencies between areas involved in classification and scales prediction: BA7 (precuneus) was involved in the stable subset of most-discriminative links as well as prediction of all five FDR surviving scales (SS35, 60, 69, 27, and 23); see also supplementary Fig. S9. Moreover, bilateral BA7 was also present in the areas involved in significantly disrupted log-degrees. Primary sensory cortex (BA1, or primary motor region adjacent to BA1) was consistently observed among the stable set of links involved in prediction of all five scales as well as classification. Area BA6 (premotor cortex) was also repeatedly observed in prediction of all five scales as well as in the disrupted log-degree maps, but was not involved in the stable links subset for classification. There were also differences: for example, while thalamus (especially in the right hemisphere) was considerably involved in predictive supervoxel links and also showed altered ss-log-degrees, it was not among the areas involved in prediction of any of the (five FDR-surviving “predictable”) scales.

Curiously, links that were most important for prediction of various scales (i.e., links that had the largest coefficients in the EN model) were not among the links that were most significantly different between the patient and control group (i.e., had the smallest *p*-values based on two-sample t-tests ran on the full dataset). Supplementary Fig. S8a shows the *p*-values of the EN-selected links, for both a CV-stable subset as well as the union of the links selected in each CV data subset, for SS69 (positive formal thought disorder) as an example. The scatter plot in Supplementary Fig. S8b also shows that the mean values of the EN-selected links are not the most different between the two groups, i.e., not necessarily very far from the diagonal line. The feature selection methods in the (healthy vs. patient) classification task vs. the disease severity prediction task were, however, different as:

1. the SANS and SAPS scales are measured only in the patient group and thus a similar features ranking approach as the one used for classification (*p*-value of *t*-test) could not be used for scale prediction; and
2. the scale prediction task was regarded as regression, where the output (symptom severity) varied between 0 and 5 (vs. a binary output, {patient, healthy} in the classification task) and thus a t-test was not useful here.

Nevertheless, other filter-based approaches can be used, such as ranking the features based on the correlation between the features and the scales severity (which can be applied to both classification and scale prediction tasks). Alternatively, classification approaches with embedded feature selection can be used for discriminating between patients and healthy controls. We aim to exploit sparse classification methods further in our future work. It would be interesting to use similar feature selection approaches for both classification and scale prediction before comparing features that contribute to each task.

### Strengths and limitations

Clearly, there are multiple directions for future research. (1) We want to test the candidate model (classifier/feature set) on different schizophrenia datasets to further evaluate the proposed methodology and learning algorithms. We also aim to develop further the stability analysis to estimate the predictive features’ reproducibility rate (i.e., stability) in other datasets. In addition, given the complexity and possible subtlety in patterns discriminating between schizophrenia and healthy control subjects, as well as the large variability in patients’ symptoms, much larger datasets are necessary to generate more accurate models of schizophrenia. One of our future directions is to analyze larger-scale datasets, such as those shared on schizconnect (http://schizconnect.org). (2) While the brain areas and connections visualized here may provide useful insight into dysconnectivity patterns revealed by fMRI, the links (and corresponding areas) discussed here are only a sufficient set of features, meaning other sets of features may exist that can yield similar prediction accuracies. Indeed, it might be possible that a learner without access to these features could still produce a classifier (that necessarily uses a different set of features) with comparable prediction accuracy. An earlier paper by Rish et al.^47^ showed such a property in predicting levels of pain using fMRI data (referred to as “holographic” information representation). Haxby et al.^48^ observed a similar property in representation of the visual information in the cortex: they showed that the category of an object can still be accurately discriminated based on fMRI-induced activation patterns even after the voxels that respond maximally to that particular object category are removed.^48^ It would be interesting to test this property in schizophrenia modeling, where many assume that the whole brain network is involved.^49^ It would also be useful to relate the findings of fMRI studies (including this paper) to the abnormalities underlying the disease: blood-oxygen-level-dependent (BOLD) fMRI signals provide only an indirect measure of neural activity, which is often diluted with artifacts, such as motion and physiological noise given the low BOLD signal strength, which could lead to systematic changes in fMRI data in one population vs. another.^50^ Here we have taken multiple steps in data processing to reduce the effect of such artifacts. Nevertheless, further investigations on mechanisms at the cellular, molecular and system level are needed before reaching conclusions about the anomalies underlying the disease. There is possibility that brain functional networks in patients are influenced by psychotropic medications.^51, 52^ Unfortunately, in this dataset we could not investigate the effect of medication because the majority of patients were medicated at the time of scans (39 out of 42 patients whose medication data was available) and non-naïve to neuroleptics (34 out of 35 patients whose medication status was available); see Supplementary Table S3 for details.

Our work, described here, represents a step towards finding more reliable objective neuroimaging biomarkers for diagnosing schizophrenia, which have higher reproducibility and generalization accuracy compared to the potential “biomarkers” reported in association studies, which are typically extracted solely based on univariate statistical tests on all of the data. We were successful in predicting several symptom severity scales using sparse multivariate regression, which is likely to be extremely important in the move towards incorporating the RDoC approach to schizophrenia as a spectrum rather than a binary label.

## Materials and methods

### Dataset

Data used for this study were accessed from the FBIRN phase II fMRI dataset,^53, 54^ downloaded from Function BIRN Data Repository, Project Accession Number 2007-BDR-6UHZ1 (fbirnbdr.nbirn.net:8080/BDR). These data include structural and task-based functional MRI data for subjects with DSM IV-defined schizophrenia or schizoaffective disorder and age-matched and sex-matched healthy control subjects, recruited at multiple scanning sites; for scanner details for the sites included in this study, see Supplementary Table S1. Various clinical measurements were also provided, including ratings on the Scales for the Assessment of Positive (SAPS)^25^ and Negative Symptoms (SANS)^24^; See Keator et al.^54^ for details. All subjects had normal hearing (no more than a 25-db loss in either ear), an IQ greater than 75 as measured by the North American Adult Reading Test, and no major medical illness, previous head injury, or alcohol/substance dependence. Control subjects with a history of major neurological or psychiatric illness and subjects with schizophreniform disorder were excluded. Patients had no significant changes in their psychotropic medications in the 2 months preceding the study.

We focused on the Auditory Oddball task-related fMRI data (for details see Supplementary Information) from five different sites, which were in compliance with Institutional Review Board for this use of data. We used 95 subjects (46 patients, 49 controls) from a total of 164 subjects in the FBIRN phase II dataset based on our preprocessing and quality control criteria as described below. (See Supplementary Table S3 for subject demographics information). We used data from the second scanning session (denoted as ‘0003’ in the original data set), and excluded subjects with fewer than 4 runs in the session. With 95 subjects and 4 runs per subject, we had a total of 380 samples (instances). (The number of patients and controls per site is given in the Supplementary Table S4).

### Preprocessing

We performed routine, per subject, preprocessing steps using FSL software.^55^ Motion correction was accomplished using MCFLIRT (rigid body),^56^ followed (separately by subject) by *tCompCor denoising*,^57^ which helped further remove physiological noise and motion-related artifacts; see Supplementary Information for more details on tCompCor denoising. Next, we performed spatial smoothing (5 mm FWHH), high pass temporal filtering (cutoff = 100 s), low-pass temporal filtering (cutoff = 2.5 TR), and registration to the MNI template through the subject’s in-session T1 scan (7 and 12 degrees of freedom for each subject’s EPI to T1 and T1 to MNI 2 mm T1 template registration, respectively, with respect to a reference image of size 53 × 64 × 37 voxel size: 3.4375 × 3.4375 × 5  mm).^56^ Subjects with missing T1-weighted scans were excluded. This left a total of 26,949 brain voxels, after applying a universal mask, which was the intersection of all subjects/runs/volumes, masked by the MNI template down-sampled to the same resolution as the EPI images. Images with severe intensity dropouts (or other physical problems, such as a reverse volume orientation) were excluded through visual inspection of outlier data (i.e., data with mean intensity larger/smaller than the site’s mean ± 2 standard deviations). Subjects with at least one problematic run were excluded as we only used subjects with 4 acceptable runs. Applying an exclusion criterion of translational motion larger than the voxel size or rotation larger than 0.06 radians in any volume (out of the 137 volumes) on the remaining subjects, no subjects were excluded. (Supplementary Table S2 gives the average motion parameter per group.)

### Functional network feature evaluation: significance, stability and predictive power

Our methodology includes the following steps: (1) computing whole-brain functional networks and extracting specific network features as discussed below; then (2) evaluating these network features according to several criteria that go beyond standard statistical significance, and involve stability across data subsets, as well as generalization (prediction) accuracy in the context of multivariate predictive modeling, which involves both binary classification of schizophrenia patients vs. controls, and sparse regression approach to predicting clinical symptoms severity measured by SANS and SAPS.

### Feature extraction

In our analysis, each sample corresponds to 1 run per subject. There are 26,949 voxels and 137 brain volumes (time points) per sample, resulting into more than 3.6 million variables. Thus, dimensionality reduction (perhaps feature extraction) is useful prior to learning a predictive model. Here, we focus on features extracted from functional brain networks,^19, 58^ which compress the data across the time-dimension, while keeping the spatial dimension. For each subject, and each run, we constructed a separate functional network and compute two main types of network features:

#### Voxel-level log-degree

For each pair of nodes (voxels), we compute pair-wise Pearson correlation coefficients among all pairs of time-series (BOLD signals); then we include a link between a pair of voxels in the network, if the correlation between the corresponding voxels’ BOLD signals exceeds a specified threshold; here, we used the same threshold of c(Pearson) = 0.7 for all voxel pairs. The degree of a node in the network is the total number of links connected to that node. We then converted the degrees into the logarithmic space, log_10_(degree + 1); the transformed features yielded better results; see below.

#### Supervoxel-level link-weights

We also extract functional brain networks for the spatially down-sampled fMRI data, in which every block of 48 (4 × 4 × 3) adjacent voxels forms a ‘supervoxel’ (size: 13.75 × 13.75 × 15 × mm) whose time-series is interpolated from the constituting voxels’ time series. This reduces the number of spatial locations to only 569 (vs. 26,949 at the voxel level), allowing the use of all pair-wise correlations across the brain: a total of 161,596 (vs. over 360 million correlations at the voxel level).

#### Within-site standardization

To account for the variance between different sites, we z-transformed every feature within each site before combining the samples from different sites (see also Supplementary Information). The resulting features are denoted as ss-log-degree, and ss-link-weights.

### Mass-univariate statistical hypothesis testing

For each single feature of a given type (e.g., ss-log-degree of each voxel, and ss-link-weight of each network link), we performed a two-sample *t*-test and ranked the features of a given type by their *p*-values, where lower *p*-values are associated with higher significance of the corresponding features. Since we need to perform one *t*-test for each of the features, which is extremely large (26,949 degree features and 161,596 link-weight features), we corrected for multiple comparisons to control the FP rate. We tried both the FDR and the Bonferroni corrections, both with the FP rate threshold *α* = *0.05*. (Variance of the *z*-transformed features were similar across the two group: ${\bar \sigma _{\rm{H}}}$ = 1.02 ± 0.037 S.D. vs. ${\bar \sigma _P}$ = 0.97 ± 0.037 S.D. for ss-log-degree and ${\bar \sigma _{\rm{H}}}$ = 0.98 ± 0.036 S.D. vs. ${\bar \sigma _P}$ = 0.99 ± 0.044 S.D. for ss-link-weights, in the healthy vs. patient group, respectively. $\bar \sigma$ denotes the average standard deviation of all features’ standard deviations. Also, the majority of features were normally distributed within each group, according to Kolmogrov–Smirnov tests ran on each feature individually.)

### Feature subset selection

To reduce the risk of overfitting, for each experiment, we first performed feature selection using a simple filter-based approach. For the classification task, we ranked the features using a function that measures the relevance of the feature to the class label (here schizophrenic patient vs. control subject), and thus the discriminative ability of the feature, and then selected the *k* top-ranking features constituting a *k*-size feature subset. We used the *p*-value resulting from the two-sample *t*-test as a ranking function, with the null-hypothesis that the feature’s values corresponding to samples of different classes came from distributions with respectively equal means and variances (assuming the variables have Gaussian distributions). *t*-test-based ranking was performed separately on each CV fold (i.e., for each training data subset selected during *k*-fold CV) in order to avoid “double-dipping” into test data during feature selection. While such filter-based approaches do not necessarily select the most-predictive subset of features,^3^ they provide a computationally efficient alternative to an otherwise computationally intractable search for the most-predictive *subset* of *k* variables. The variables were then ranked in the ascending order of their *p*-values, with the lower ranks indicating more informative/relevant features.

For the scale prediction task, we used an embedded feature selection approach, in which relevant features were selected during learning a regression model on the data (see Supplementary Information), rather than as a preprocessing step as in filter-based feature-selection approaches. Note that, unlike the classification task (patient vs. healthy control), scale prediction was a regression problem, each scale’s values are in the range between 0 and 5, with the larger scores representing more severe symptoms. Thus, a regression approach with inherent feature selection was used for modeling the symptom severity scales (the Discussion discusses other possible approaches for scale prediction as well as classification).

### Feature stability analysis

The goal of this analysis was to assess the stability of the features selected across various subsets of the data (i.e., sets of samples). For this, we generated 95 data subsets, leaving one subject out in each fold (this includes four samples corresponding to the 4 runs per subject). The feature selection procedure was then performed in each fold for various-size feature subsets. The stability analysis determined what fraction of the features in each feature subset was common across the folds (for the embedded feature selection approach, used in the scale prediction task, feature selection occurred as an embedded part of the EN classifier training for various size feature subsets).

### Classification: schizophrenic patients vs. controls

A series of classifiers was then trained using various-size feature subsets (as input), to discriminate between patients and healthy controls (class labels, {patient, control}, as output). The process was repeated for each feature type separately: first ss-log-degree then ss-link-weights. Various classifiers were considered for the patient vs. control classification task: nearest neighbors, linear SVM, RBF SVM, decision tree, random forests, logistic regression, GNB, and linear discriminant analysis; see Supplementary Information for tuning of hyperparameters. Each of the models’ (classifier/feature set) were evaluated using leave-one-subject-out CV; see below.

### Predicting disease symptom severity

Next, we experimented with the scale prediction task. Following previous studies,^18^ univariate correlations were first computed between link-weight features and each of the rating scales reflecting the severity of schizophrenia. Nine Global Rating Scales were used, from the negative (SANS) and positive (SAPS) symptom scales (each involving scores for a total of 24 and 34 different scales, respectively) provided for patients. (A list of the Global Rating Scales and their distribution in the patient group—demonstrating the heterogeneity of disease symptoms in the patient group—is presented in Supplementary Fig. S1.)

In contrast to previous work, our objective was to go beyond the univariate analysis on a single dataset, and evaluate generalization accuracy of link-weight features when used by multivariate regression models (each scale was modeled separately) to predict the scales of previously unseen subjects; here we used the EN sparse linear regression model^59^; see Supplementary Information for more details on the EN model.

### Evaluation via CV

For each predictive model, whether it was a classifier on a feature subset of a certain size, or a regression model, we computed an estimate of model’s generalization error to previously unseen data using *k*-fold CV approach. We used leave-one-subject-out CV to evaluate each model’s performance: we generated *S* = 95 train/test set combinations of the samples (CV-folds), where each of the folds would set aside as a test set the four samples for a particular subject (we used *S* = 46 for disease symptom severity prediction). Then, we learned a model on the remaining samples, where the learning process involved both feature ranking and subset selection. The model is then tested on the test set involving four samples corresponding to the left-out-subject.

To avoid a biased estimate of generalization error, as mentioned above, variable selection was performed separately on each CV training dataset. Indeed, performing variable selection on the full dataset would constitute “double-dipping” producing overly optimistic results, since the learning process should use absolutely no information about the test data labels during the training phase, which also includes variable selection.

### Data availability

Data used for this study were accessed from the FBIRN phase II fMRI dataset,^53, 54^ downloaded from Function BIRN Data Repository, Project Accession Number 2007-BDR-6UHZ1 (fbirnbdr.nbirn.net:8080/BDR).

### Code availability

Code for classification is available upon request.

## Electronic supplementary material

Supplemental Material
