# Supplementary material for: Learning stable and predictive network-based patterns of schizophrenia and its clinical symptoms
Source: NPJ Schizophr. 2017 May 16;3:22. doi: 10.1038/s41537-017-0022-8 (PMC5441570; doi:10.1038/s41537-017-0022-8)
Supplement: Supplementary file 1 — Supplemental Material [file 41537_2017_22_MOESM1_ESM.doc]

**Supplementary Information**

**Learning Stable and Predictive Network-based Patterns of Schizophrenia and its Clinical Symptoms**

Mina Gheiratmand, Irina Rish*, Guillermo A Cecchi, Matthew R G Brown, Russell Greiner, Pablo I Polosecki, Pouya Bashivan, Andrew J Greenshaw, Rajamannar Ramasubbu, Serdar M Dursun

^*^*Correspondence*: *rish@us.ibm.com*

Pages: 29; Figures: 9; Tables: 10; Appendix: 3 Figures.

**Supplementary Figures**

Figure S1. Histogram of symptom severity in patient samples.

Figure S2. FDR plots for ss-link-weights and ss-log-degrees.

Figure S3. Scatter plot of average degree per voxel in patients vs controls.

Figure S4. Total error, FN and FP rates for ss-link-weight and ss-log-degree.

Figure S5. Scatter plot of average link-weight in patients vs controls for all links..

Figure S6. Predictive accuracy of the EN for the scale SS35, attention.

Figure S7. Stable subset of links for predicting SS35.

Figure S8. Statistical significant of the CV-stable EN-selected link-weight features.

Figure S9. Summary of all areas involved in classification and scale prediction.

**Supplementary Tables**

Table S1. Scanner and image acquisition information per site.

Table S2. Motion parameters.

Table S3. Subjects demographic information.

Table S4. Subject combination per site.

Table S5. Summary of the largest most-significant clusters (ss-log-degrees).

Table S6. Lowest CV error rates for various classifiers using ss-link-weights.

Table S7. Summary of nodes involved in the most-discriminative stable links.

Table S8. Summary of nodes involved in prediction of specific scales.

Table S9. Summary of the stable predictive links involved in classification.

Table S10. Summary of stable links involved in prediction of specific symptom scales.

**Appendix**

Figure A1. Predictive accuracy of the EN model for nine global rating scales.

Figure A2. Stability of features selected by the *best* EN model.

Figure A3. Coefficients of features in the *best* EN model averaged over CV-folds.

**Auditory Oddball Task**

In this task, the subjects pressed a button when they heard an oddball tone (1200 Hz) presented infrequently in 5% of the trails (i.e. every 6-15 s) while focusing on the fixation cross in the middle of the grey screen. In all other trials, a standard tone (1000 Hz) was presented (inter-stimulus interval = 500 ms; trial duration = 100 ms). There were 4 runs per scanning session, each taking 280 s. The functional scans were T2*-weighted gradient echo EPI sequences, with TR = 2, TE = 30 ms, flip angle 90, acquisition matrix 64 x 64, 22 cm FOV, 27 slices when possible, 4 mm thick with 1 mm gap, oblique axial AC-PC aligned, and 140 TRs.

**Preprocessing – tCompCor denoising**

For each subject, the tCompCor^1^ algorithm regressed out, from every voxel, the top six principal components extracted from the time-series of the top 2% of the voxels identified as the most “noisy”, based on the (high) variance in their time-series. The slow drift in time-series was removed before computation of the variance. As expected, noisy voxels were located primarily at the brain edges and ventricles, suggesting that the *tCompCor* denoising step removed physiological noise and motion-related artifacts

**Within-site feature standardization**

Our data came from 5 different sites, four of which provided between 21 and 23 subjects (88-92 samples per site) and one provided 6 subjects (24 samples). To account for the variance between different sites, we z-transformed every feature *i* within each site *j* (*x_ij_*) before combining the samples from different sites, using the empirical mean (*μ_ij_*) and standard deviation (*σ_ij_*) of that feature across the samples within site *j:*

**Tuning of hyperparameters**

Hyperparameters were selected based on a grid search over the parameter space and optimized (with respect to accuracy) via an internal 5-fold cross validation. For classifiers with larger than 10 parameter settings (e.g. RBF SVM) a randomized search with 10 iterations was used. Parameter space for different classifiers were as follows: RBF SVM: C: {0.01, 0.1, 1.0, 10.0, 100.0}, gamma: {0.01, 0.025, 0.063, 0.158, 0.398, 1.0, 1.778, 3.162, 5.623, 10.0}; Logistic Regression: C: {1.3, 3.8, 11.7, 35.5, 108.0, 328.6, 1000.0}; Random Forest: max_depth: {2, 3, 5, 7, 10, 16}; Nearest Neighbors: n_neighbors: {1, 5, 10, 20}. For Linear SVM, the default C = 1 was used. For LDA, Decision Tree and Naïve Bayes classifiers, no hyperparameter tuning was involved.

**Sparse regression model: the Elastic Net (EN)**

The Elastic Net is a regularized linear regression model, where the regression coefficients are penalized according to a linear combination of their l1- and l2-norm. Parameters λ_1_ and λ_2_ denote the weight of l_1_- and l_2b_-norm regularization, respectively:

*L*(*β|λ_1_ ,λ_2_*) = ${\left| \mathbf{|}\boldsymbol{y}-\boldsymbol{X}\beta\right||}_{2}^{2}+\lambda_{1}{||\beta||}_{1}+ \lambda_{2}{||\beta||}_{2}^{2}$

is equivalent to the ridge regression.

The l_1_-norm enforces the *sparsity* of regression coefficients, i.e. leaves only a relatively small number of non-zero coefficients, which is controlled by the corresponding sparsity parameter λ_1._ The l_2_-norm constraint ensures the grouping property over correlated variables: similar coefficients are assigned to predictors that are highly correlated with each other, thus including or excluding them together, the property which l_1_-norm constraint alone is not providing. This can improve the interpretability of the model, for example, including a group of similarly relevant voxels, rather than one representative voxel from the group. Note that EN becomes equivalent to LASSO when $\lambda_{2}=0$ and $\lambda_{1}>0,$ while for $\lambda_{1}=0$and $\lambda_{2}>0$ it is equivalent to the ridge regression. Herein, we use the EN regression for predicting scales measuring the severity of symptoms. (Note: X is normalized and Y is centered for use in the EN model.) We used an implementation of the EN algorithm in Matlab by Karl Skoglund, downloaded from <http://www2.imm.dtu.dk/pubdb/views/publication_details.php?id=3897> in 2008.

We evaluated the Elastic Net for three different values of its parameter λ_2_. (The smaller the λ_2_ value, the closer the EN model is to the LASSO, the l_1_-norm regularized linear regression; EN with λ_2_=0 is equivalent to the LASSO.) For a fixed λ_2_, the model is tested for five different levels of sparsity, i.e. the number of nonzero coefficients, with maximum of 700 nonzeros. We used, again, leave-one-subject-out cross-validation – i.e., we trained the model on all but one subject and predicted the scales for the left-out subject, repeating this procedure for each of the schizophrenic subjects. Spearman’s Rho correlation coefficient was then found between the predicted and actual scales on all subjects (46 subjects x 4 runs = 184 samples).

For the scales surviving FDR, the best prediction was achieved with the EN model using the following parameters λ_1_ (measured by the number of nonzeros, i.e. features selected) and λ_2_: for SS23, 100 features, λ_2_ = 10; for SS27, 300 features, λ_2_ = 10; for SS35, 700 features, λ_2_ = 1; for SS60, 50 features, λ_2_ = 10, for SS69, 50 features, λ_2_ = 10. (The EN model prediction for SS35 and all the EN model parameter settings is shown in Supplementary Figure S6.) EN prediction, as well as the average predictor coefficients and predictors stability for all 9 scales are shown in three separate figures in the Appendix. (In Figure 5, we visualize the model with the lowest number of nonzero coefficients that had a significant correlation within 5% of the highest correlation. For scale SS35, this results in an EN model with 300 features and λ_2_ = 0.1.)

**Scale Prediction Results – Global ratings of Alogia (SS23) and Avolition-Apathy (SS27)**

Maps of the stable predictive links for the two remaining scales that survived FDR correction, SS23 and SS27, are given in the Supplementary Figure S7. Stable links for the prediction of SS27 (avolition/apathy) (42 links out of 300 links) included several bilateral connections between Brodmann areas 21 and 22, with some nodes adjacent to the Primary Auditory area (BA41), as well as several bilateral connections between Brodmann areas 40 (and Supramarginal Gyrus) including the two strongest links, shown as the thickest links in the supplementary Figure S7d.

Stable links for prediction of SS23 included 11 links (out of 100) with the most significant links involving Cerebellum.L-Primary Sensory (BA1).L and BA7.R-Supramarginal Gyrus (SMG).R, and several connections (6 out of 11) spanning across the two hemispheres, which involved Brodmann areas 6, 8, 47, 19, and cortical areas Middle Temporal Gyrus (MTG).L and Lateral Occipital Cortex (LOC).R.

**Supplementary Figures**

| SANS |  |
| --- | --- |
| SS17 | Global Rating of Affective Flattening |
| SS23 | Global Rating of Alogia |
| SS27 | Global Rating of Avolition-Apathy |
| SS32 | Global Rating of Anhedonia-Asociality |
| SS35 | Global Rating of Attention |
| SAPS |  |
| SS42 | Global Rating of Severity of Hallucinations |
| SS55 | Global Rating of Severity of Delusions |
| SS60 | Global Rating of Severity of Bizarre Behavior |
| SS69 | Global Rating of Positive Formal Thought Disorder |

**Figure S1.** Histogram of symptom severity in patients (S = 46). Nine Global Rating scales for negative symptoms (SANS) and severity of positive symptoms (SAPS). (FTD: formal thought disorder)

**a**

**b**

**Figure S2.** FDR plots for (a) ss-link-weights and (b) ss-log-degrees. (ss stands for site-standardized, i.e. each feature is standardized based on the samples within each site before combining the samples from five different sites.)

**Figure S3.** Mean degree per voxel (n = 26,949) in patients (y-axis) vs controls (x-axis). The average degree value is larger in the patient group vs. healthy controls for the majority of the voxels. The bonferroni-survivng most-significant voxels, identified using the whole dataset, are marked in green crosses and are the farthest from the diagonal line. The red circles show the cross-validtion-stable Bonferroni-sruviving voxels based on the standardized log-degree values. These are the voxels that are displayed on a brain map in Figure 1 of the main text.

**a**

| **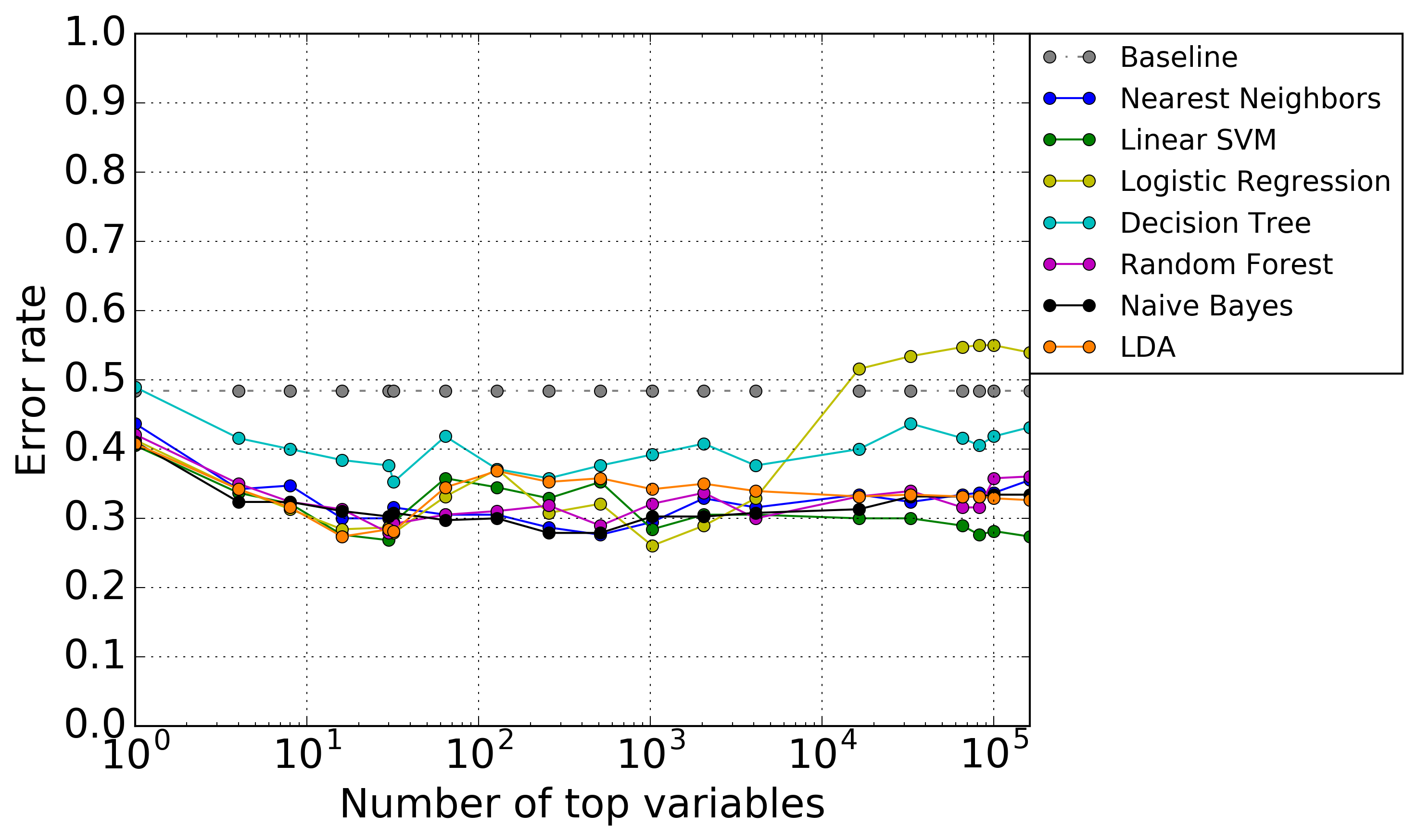** | **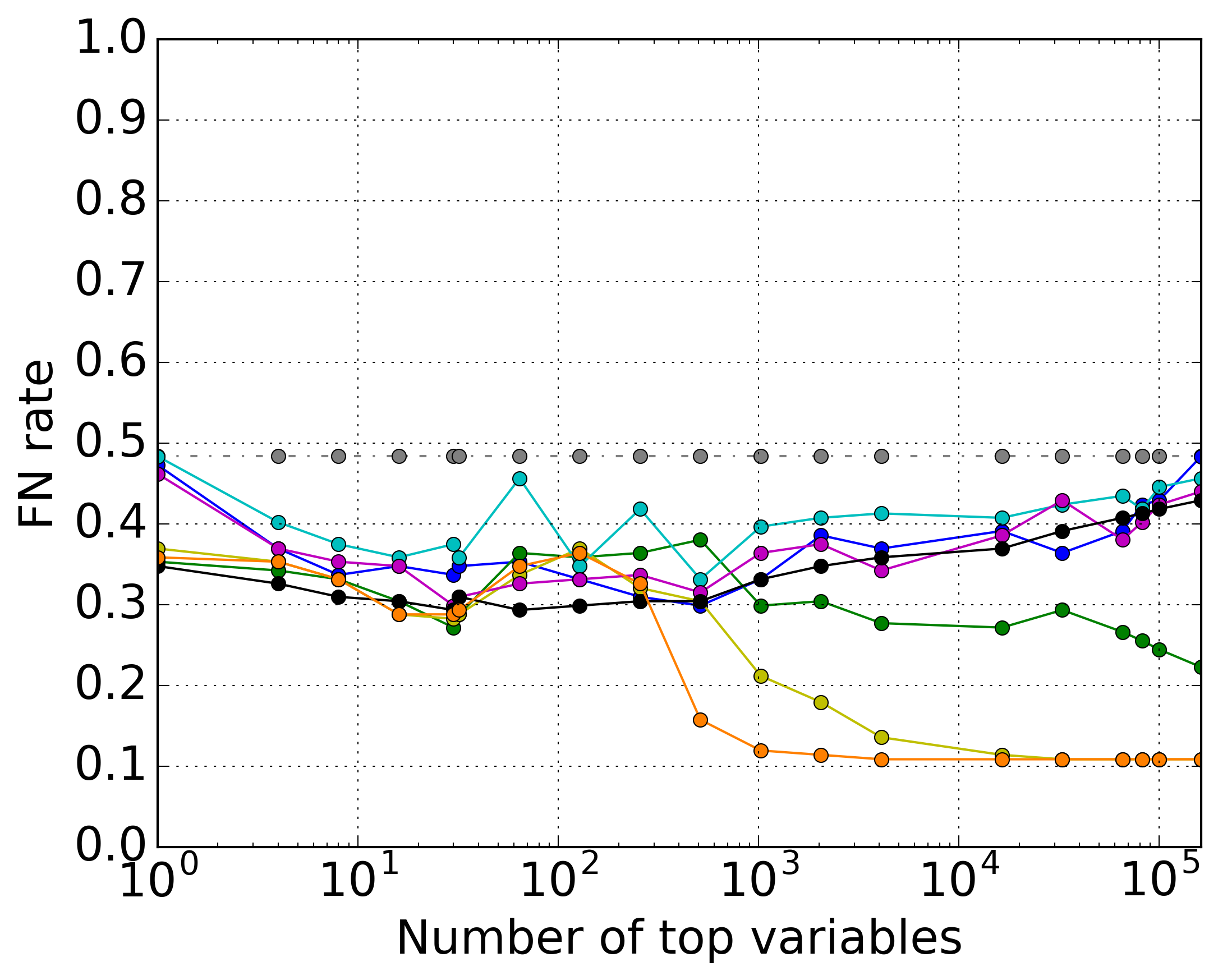** |
| --- | --- |
|  | **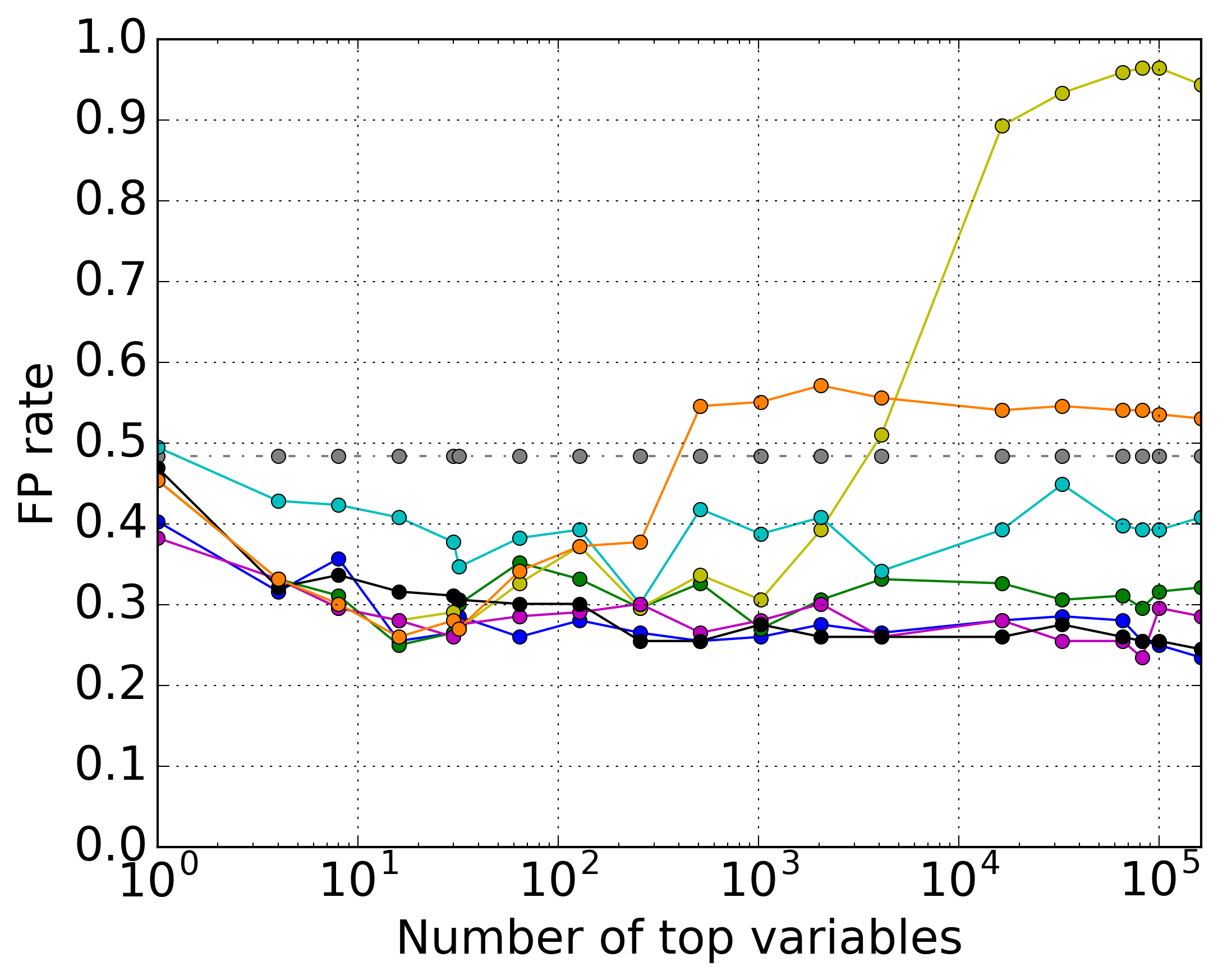** |

**b**

| **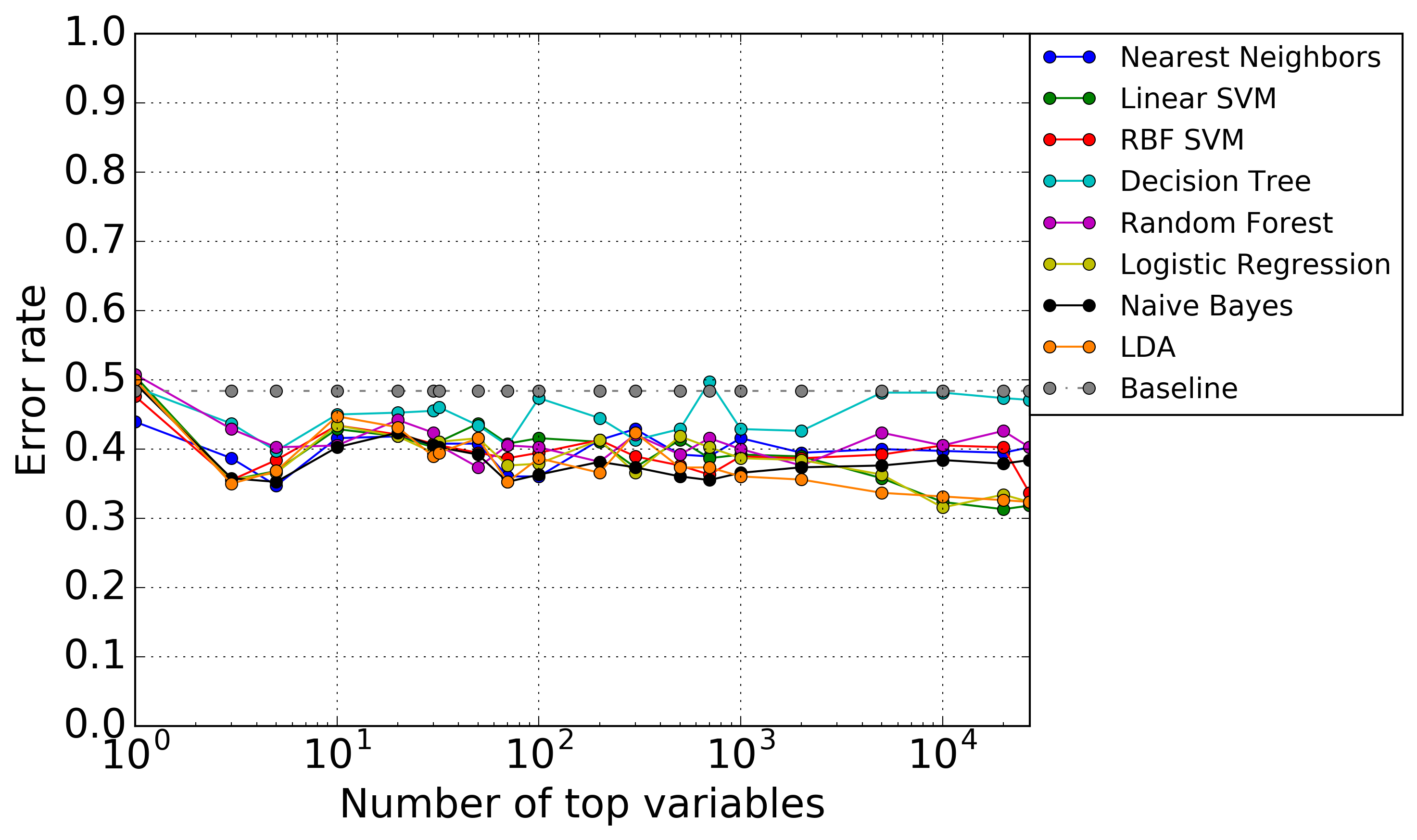** | **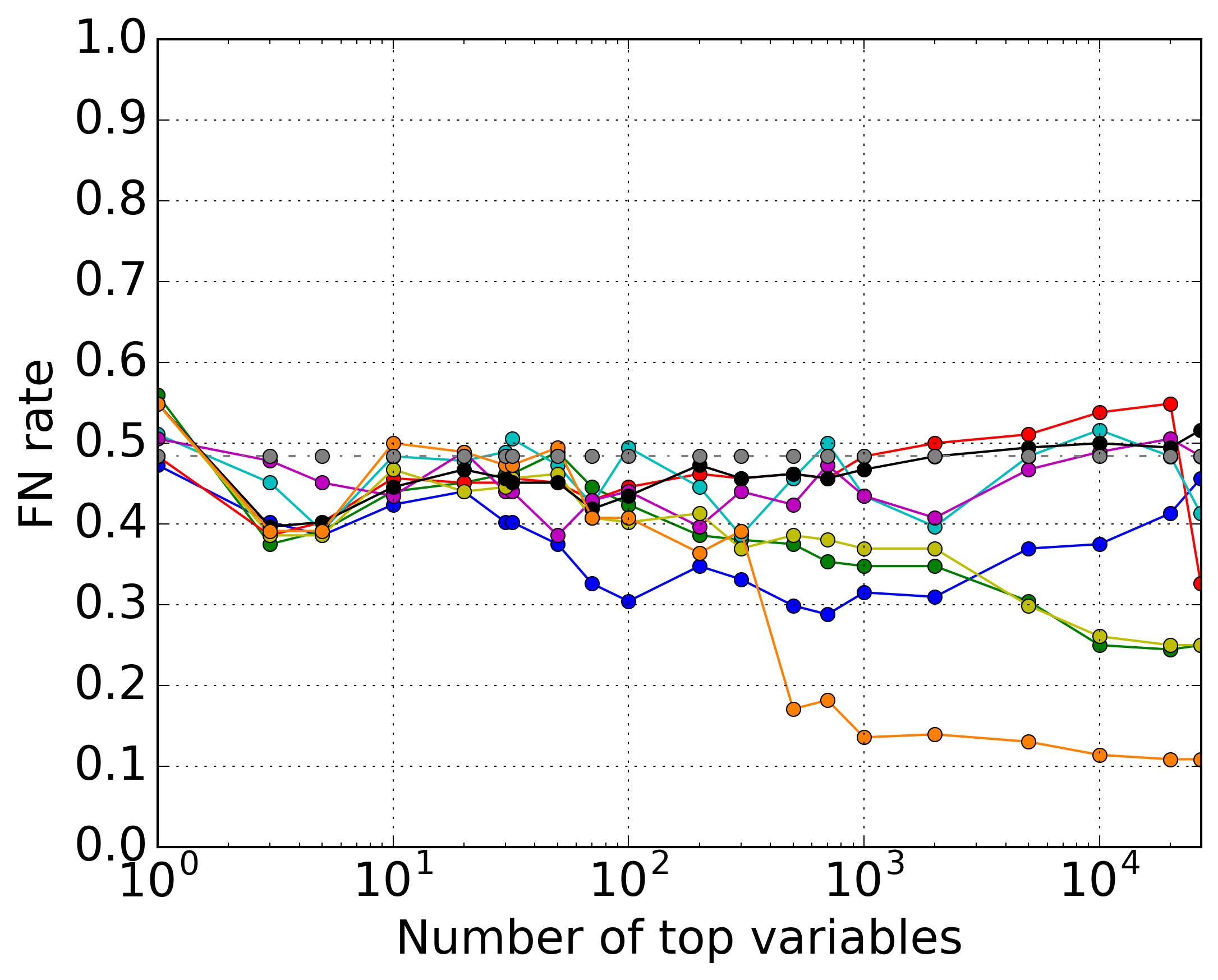** |
| --- | --- |
|  | **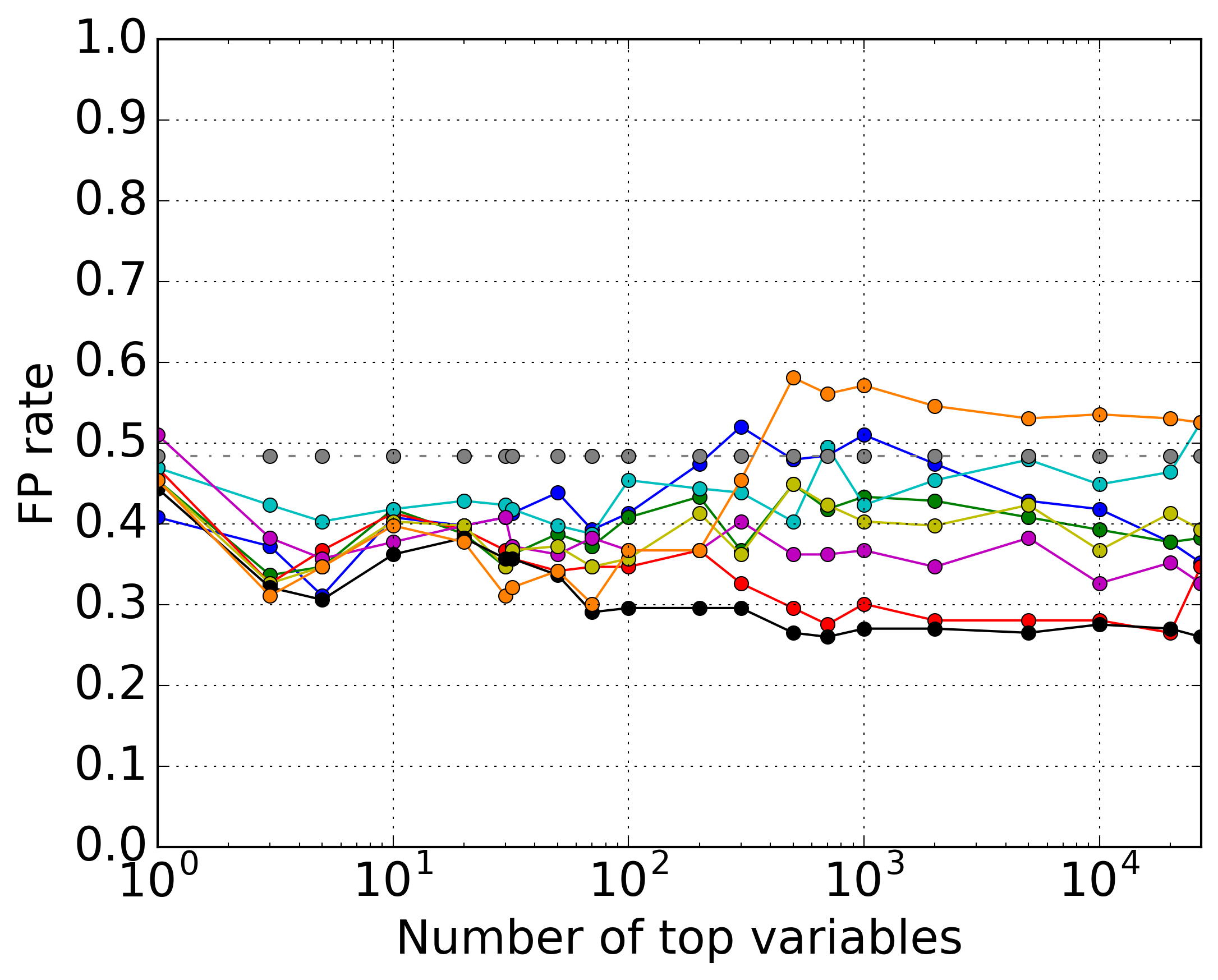** |

**Figure S4.** Total error, FN and FP for classification of schizophrenia patients from healthy controls using a) ss-link-weight and b) ss-log-degree features.

**
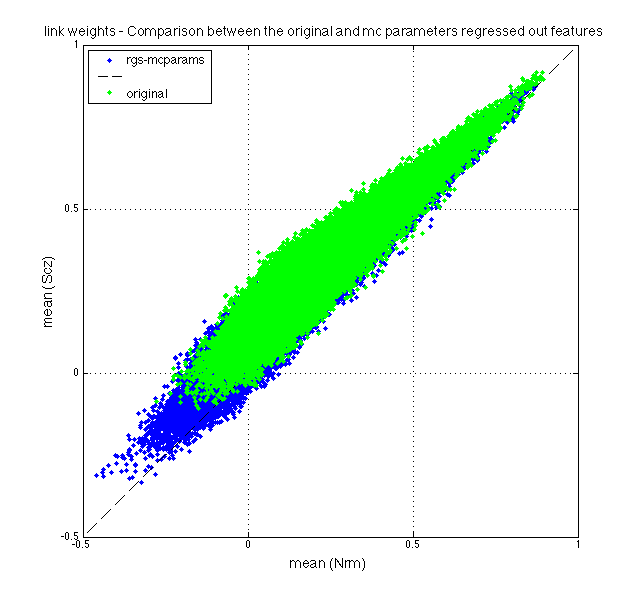
**

**Figure S5.** Link-weight increase in schizophrenia: mean link-weights, per link, within control (x-axis) versus patient (y-axis) group. Blue: before regressing out absolute and relative frame-to-frame motion displacement parameters (provided by FSL after conducting motion correction); green: after regressing out those parameters from the link-weight features. (Features are extracted from low pass filtered *tCompCor* denoised fMRI data.) The graph shows that the unidirectional effect, i.e. increased correlations in the patient group, remains after regressing out the two motion parameters.

**Figure S6.** Predictive accuracy of the Elastic Net for the scale SS35, global rating of attention, measured by Spearman correlation between the actual and predicted scale values, for a range of sparsity parameter (x-axis, number of variables selected) and grouping parameter λ_2_. The grey plots show the average correlation +/- S.D. in the training data (EN model prediction for all other scales is given in the Appendix section at the end of the Supplementary Material.)

| **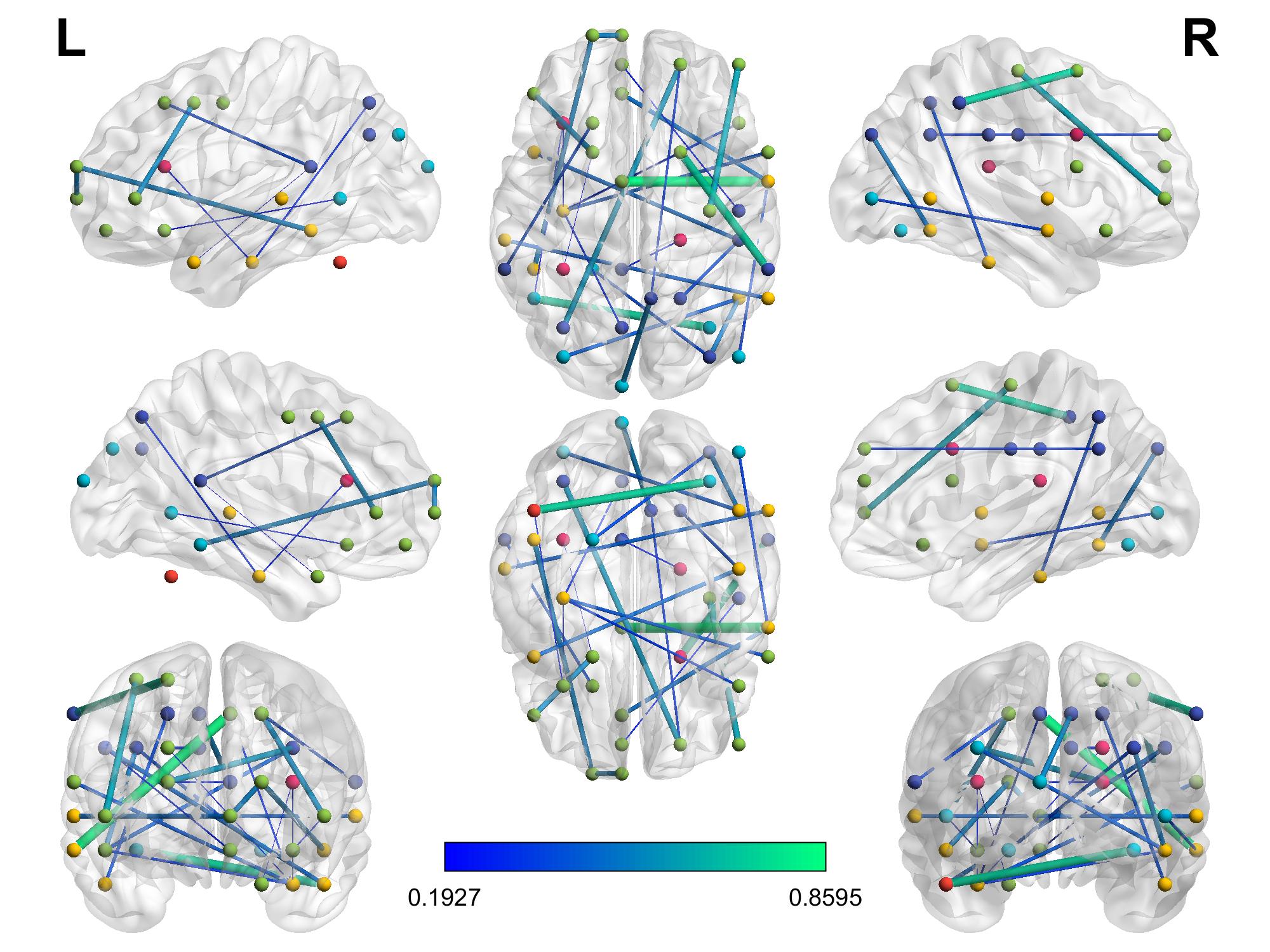** | **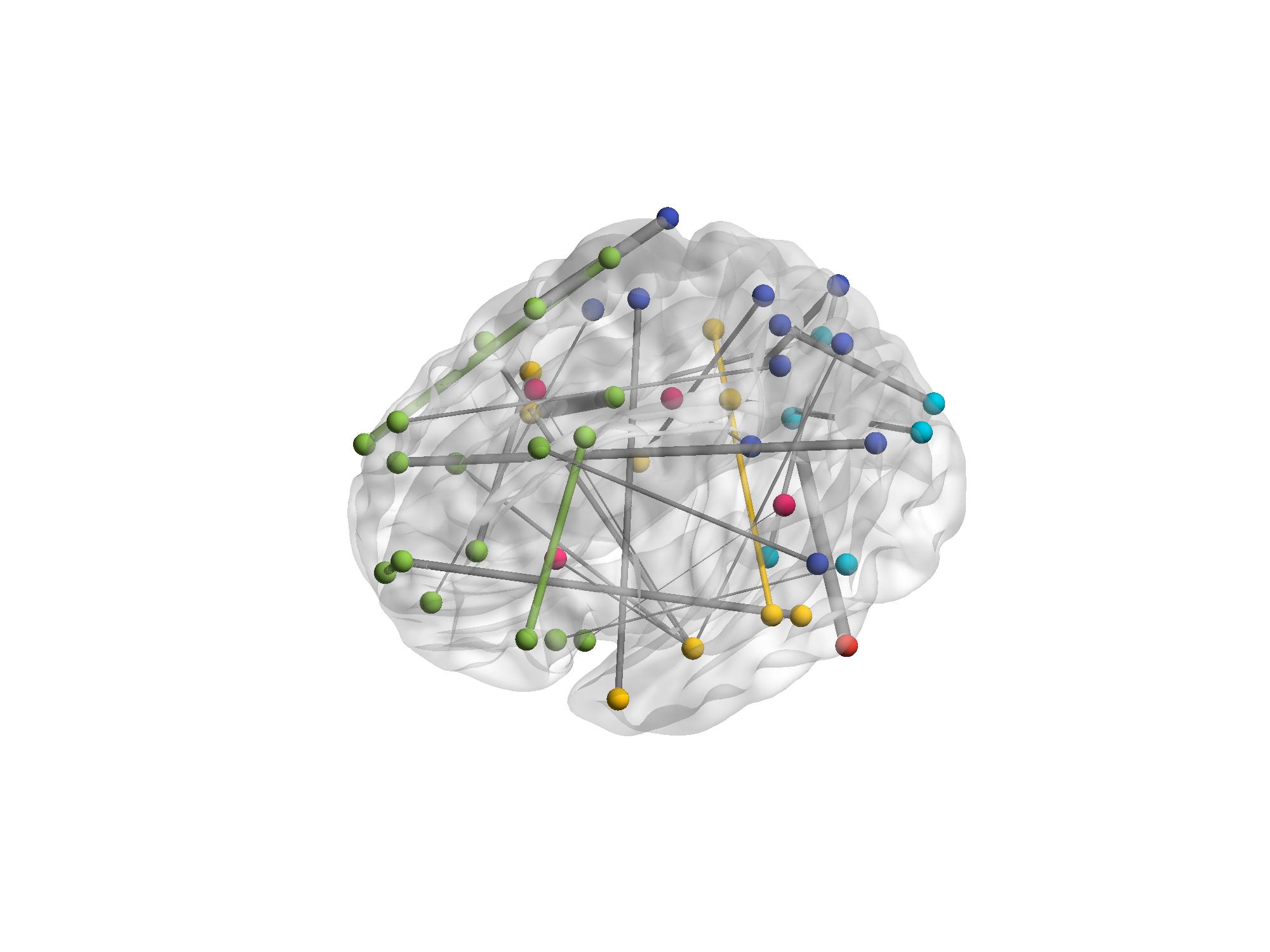** |
| --- | --- |
|  | **** |

**
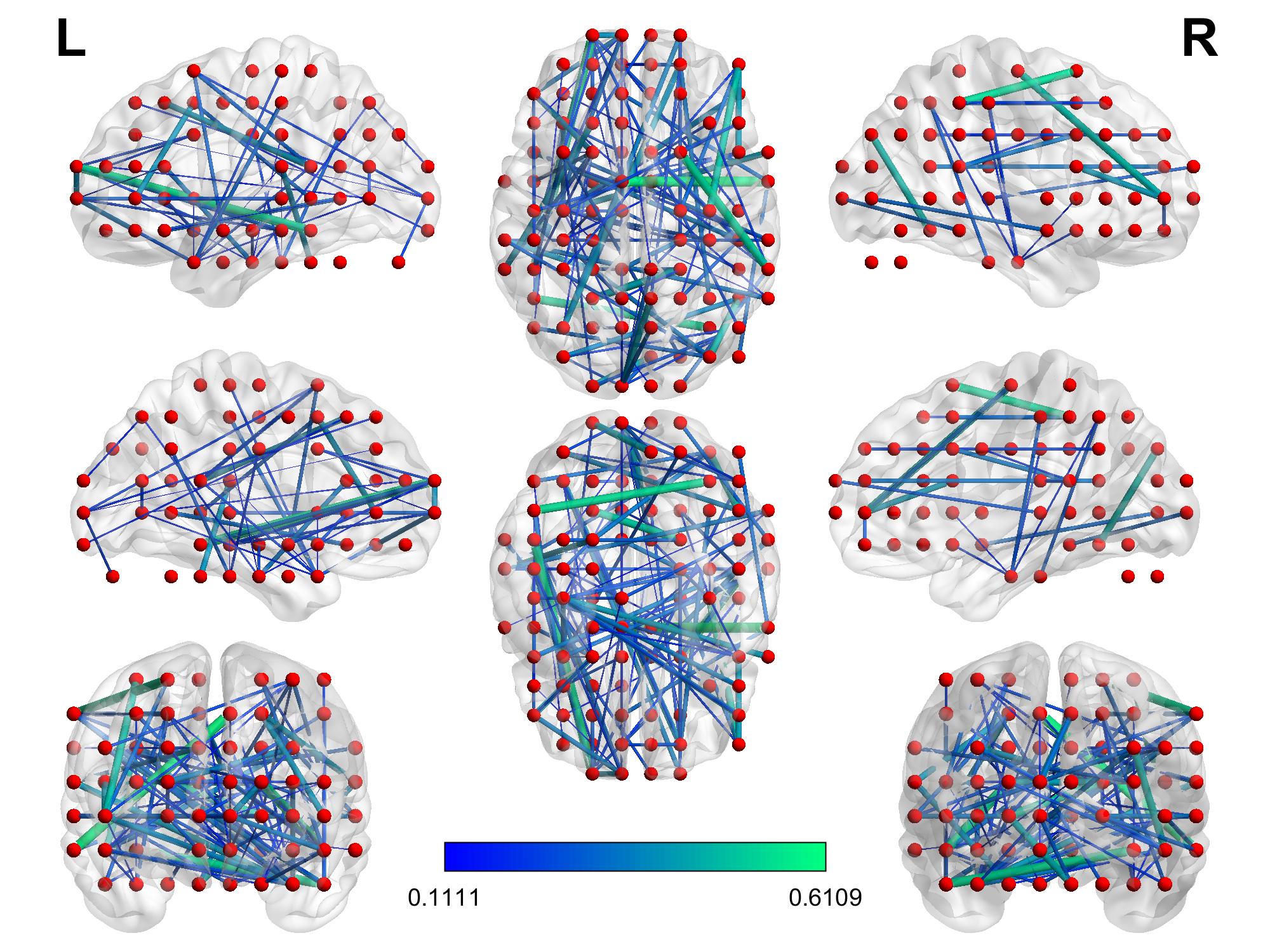
**

BA label acronyms in the labeled links map in the upper panel: FG: Fusiform. Anatomical label of nodes with BA labels: BA47: FOC; BA24.L: Cingulate Gyrus, anterior division; BA45.L: Frontal pole; BA23.L: Cingulate Gyrus, posterior division). Nodes for which the BIS tool did not provide a label: FOC (Frontal Orbital Cortex), LOCid (Lateral Occipital Cortex, inferior division); CWM (Cerebral White Matter); PCG (Precentral Gyrus); FP (Frontal Pole); LOCsd (Lateral Occipital Cortex, superior division); SMGpd (Supramarginal Gyrus, posterior division) Anatomical labels are based on the Harvard-Oxford Structural Cortical or Subcortical Atlases.)

**­­**

**Figures S7a.** Stable subset of links for predicting scale for global rating of attention (SS35). Top panels: links corresponding to the EN model with 300 nonzero features. The top right panel shows the modular node color and connections: Most links are long range, spanning between separate lobes (grey), rather than within similar lobes (colored links, e.g. yellow and green links within the frontal and temporal lobes). (X, Y, Z coordinates of the nodes and the corresponding Brodmann area labels are summarized in Supplementary Table S8. List of all area-to-area connections are presented in Table S10.a. Bottom panel: stable links corresponding to the EN model with 700 nonzero features (the model that achieved the highest correlation; see the Results section). (For a reference map of all Brodmann areas see Supplementary Figure S9.)

**
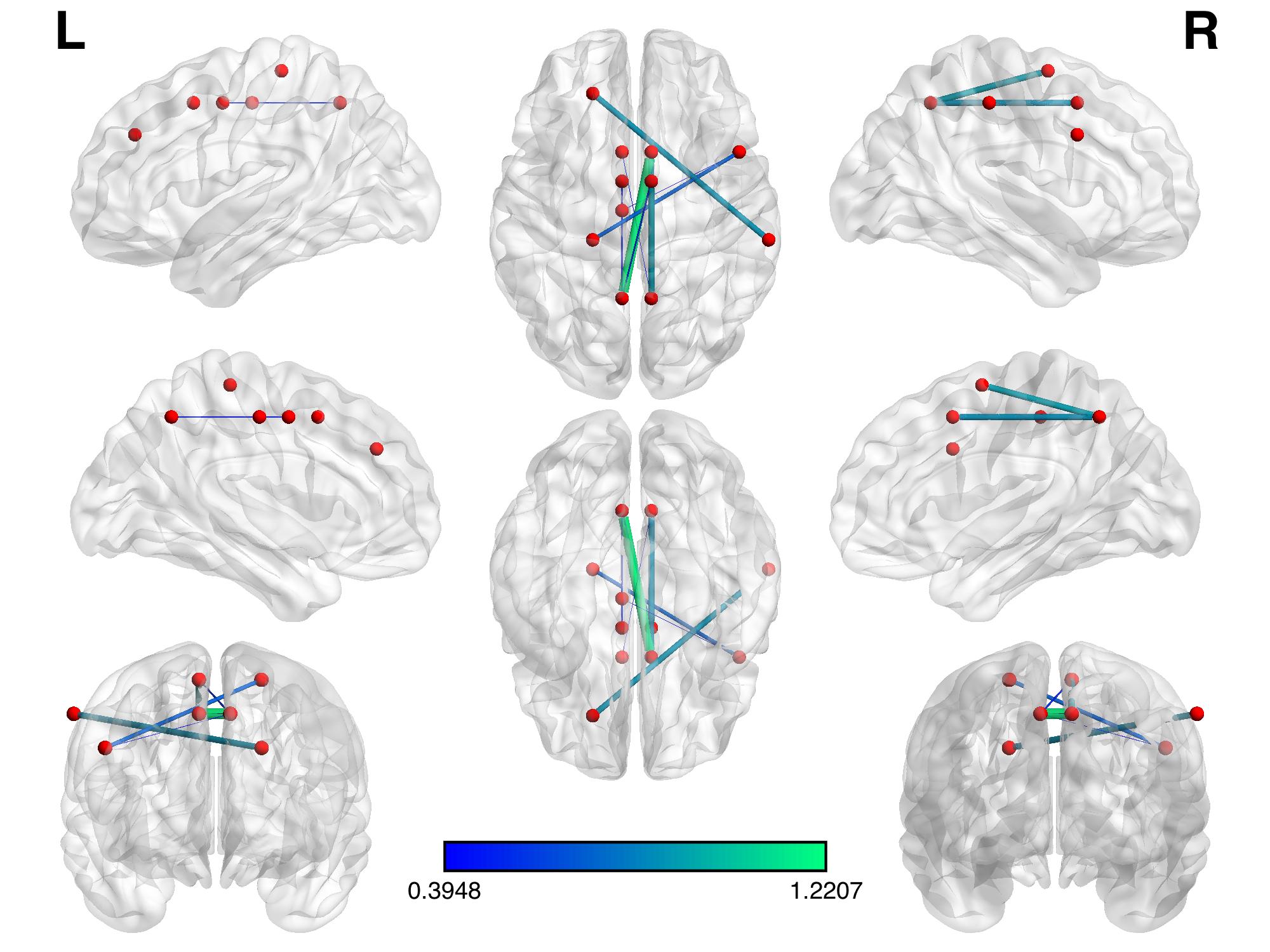
**

**Figures S7b.** Stable subset of links for predicting the scale for global rating of severity of bizarre behavior (SS60).


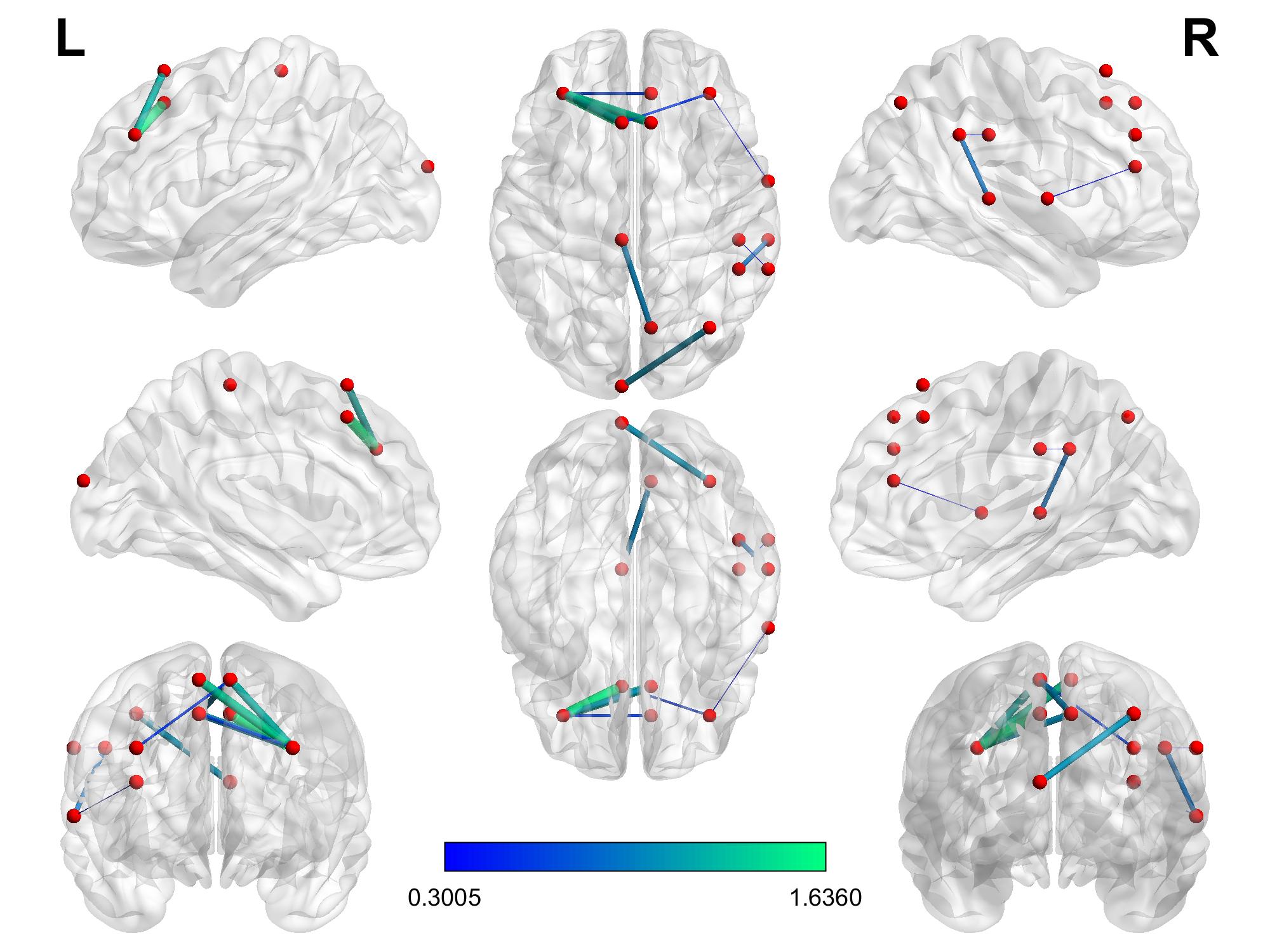


**Figures S7c.** Stable subset of links for predicting the scale for global rating of positive formal thought disorder (SS69).

**
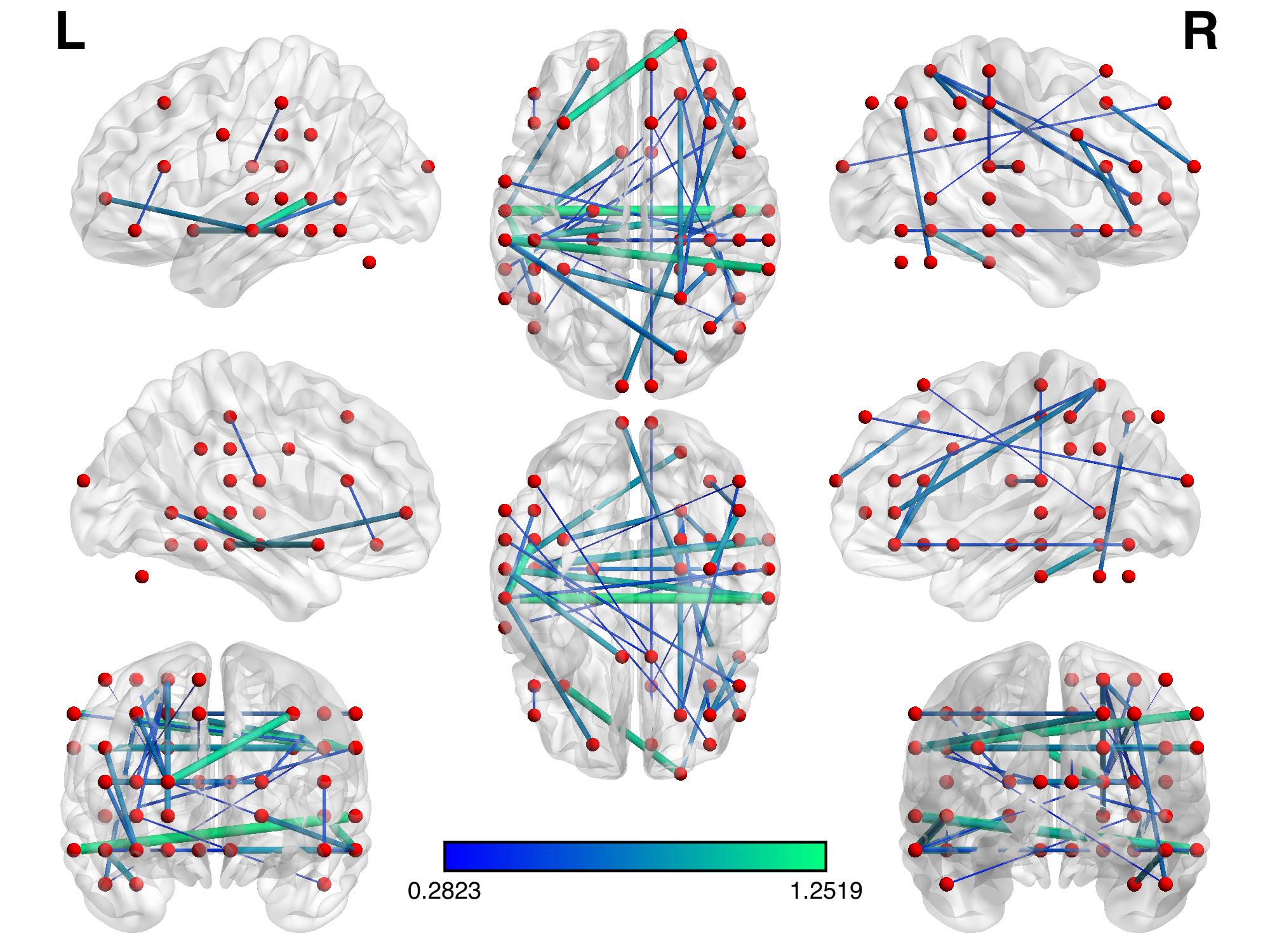
**

Acronyms (for labels based on the Harvard-Oxford Structural Atlas): OP: Occipital Pole (A similarly located node in the left hemisphere is labeled VisAssoc.R based on BIS); LOCsd: Lateral Occipital Cortex, superior division (adjacent to BA19.R); SMGpd: Supramarginal Gyrus, posterior division; FP: Frontal Pole; CWM: Cerebral White Matter.

**Figures S7d.** Stable subset of links for predicting the scale for global rating of Avolition-Apathy (SS27).

**
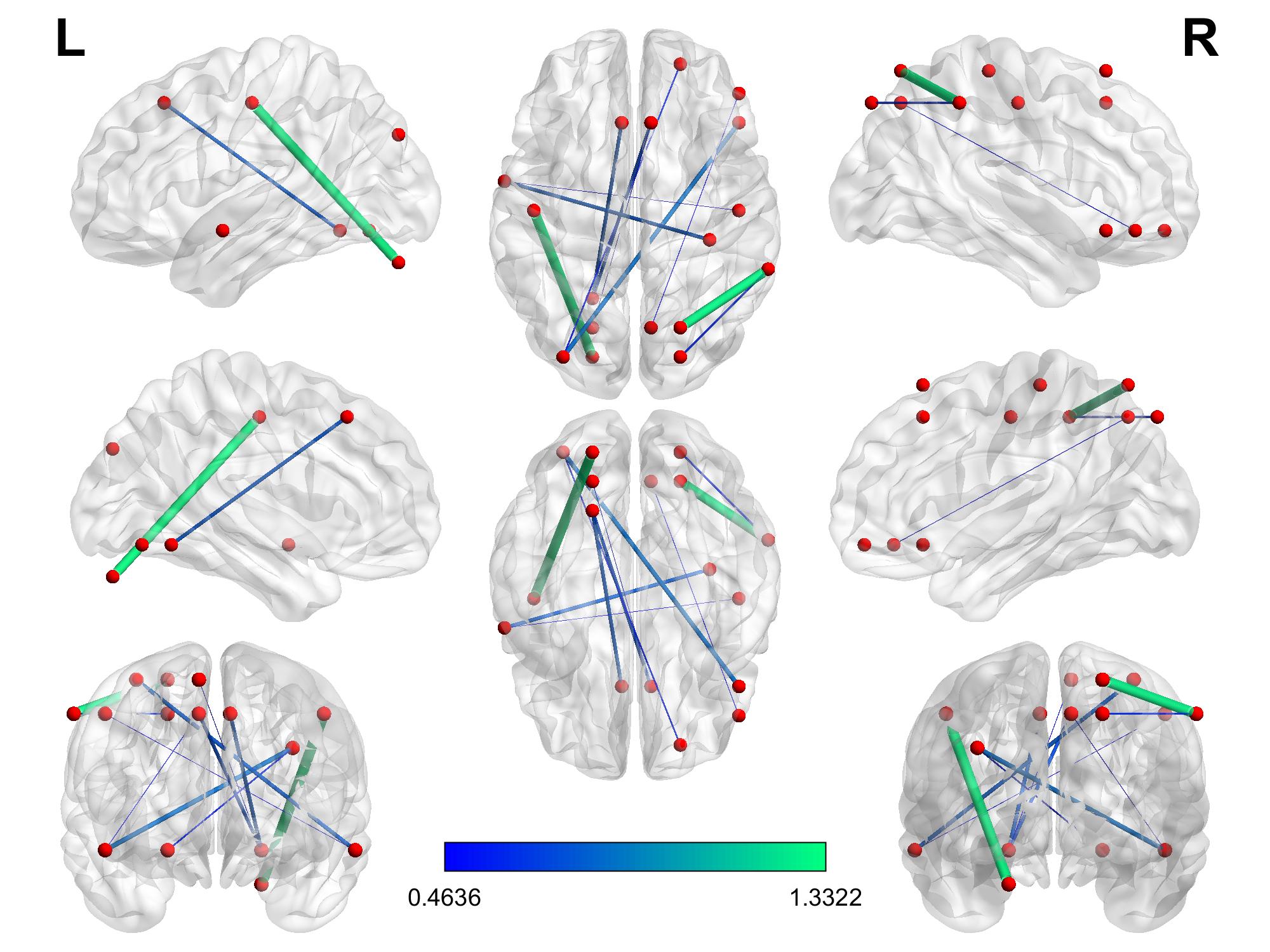

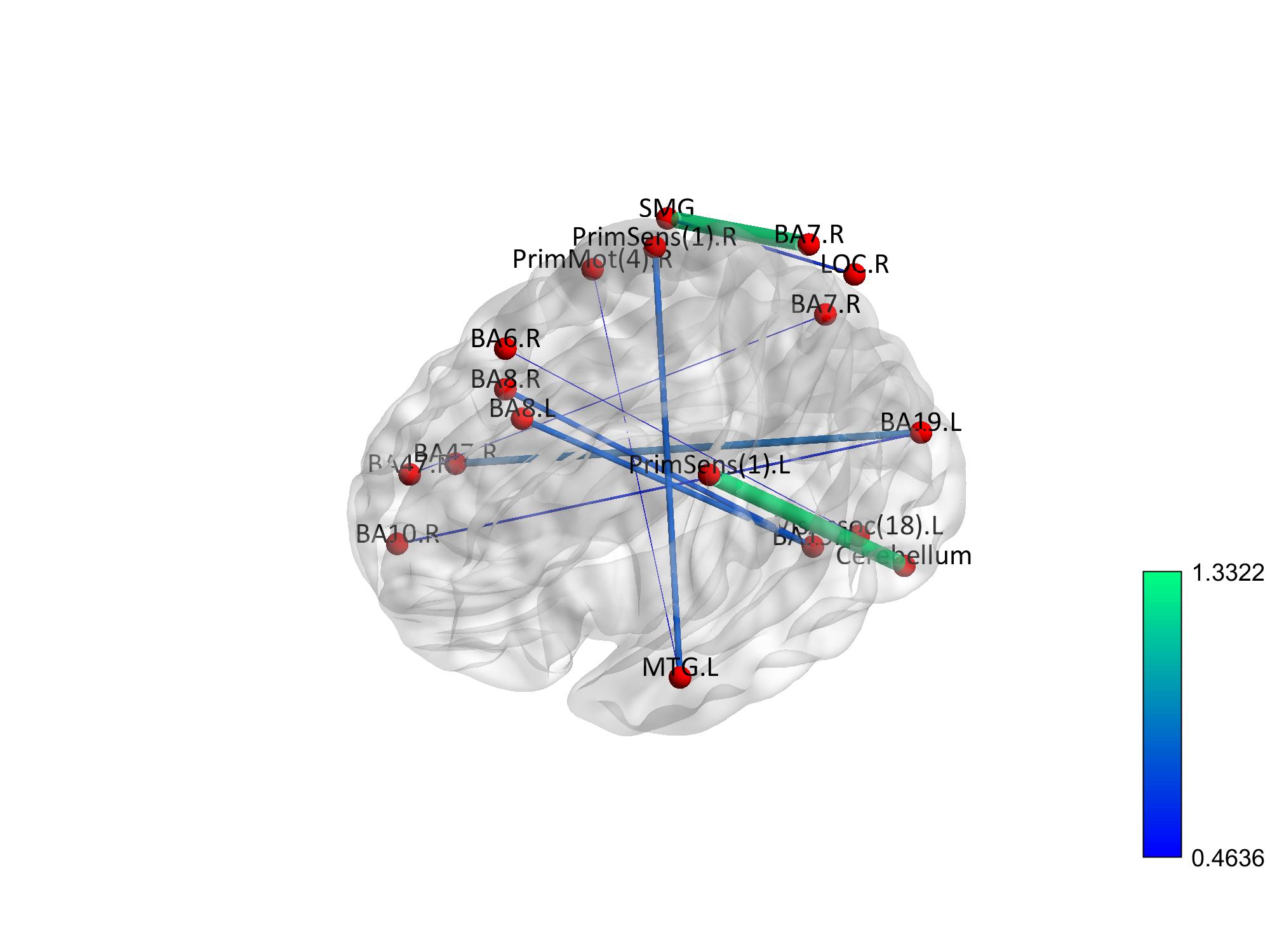
**

The labels for three out of 18 nodes were found using the fsl Harvard-Oxford atlas (fsl atlasquery) as the BIS tool did not provide Brodmann labels for those coordinates: MTG: Middle Temporal Gyrus (anterior division), LOC: Lateral Occipital Cortex, SMG (Supramarginal Gyrus, posterior division) (parietal lobe, BA 40). (Both nodes labeled as PrimSens (Primary Sensory region) are adjacent to the Primary Motor region.)

**Figures S7e.** Stable subset of links for predicting the scale for global rating of Alogia (SS23).

**a**

**b**

**
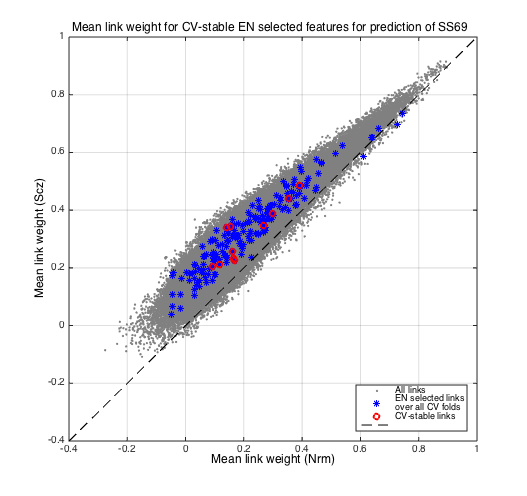
**

**Figure S8.** Statistical significance of the CV-stable EN-model-selected link-weight features for prediction of SS69 (global rating of positive formal thought disorder): a) FDR plot and b) scatter plot of the mean of each feature in the patients (y-axis) vs. healthy control (x-axis) group. The plots suggest that the CV-stable EN-selected links that contribute to predicting scales for global rating of positive formal thought disorder in patients are not necessarily among the most-significant features between the healthy and patient group. (The results for the other 4 FDR surviving scales is similar and is not shown here.)

**
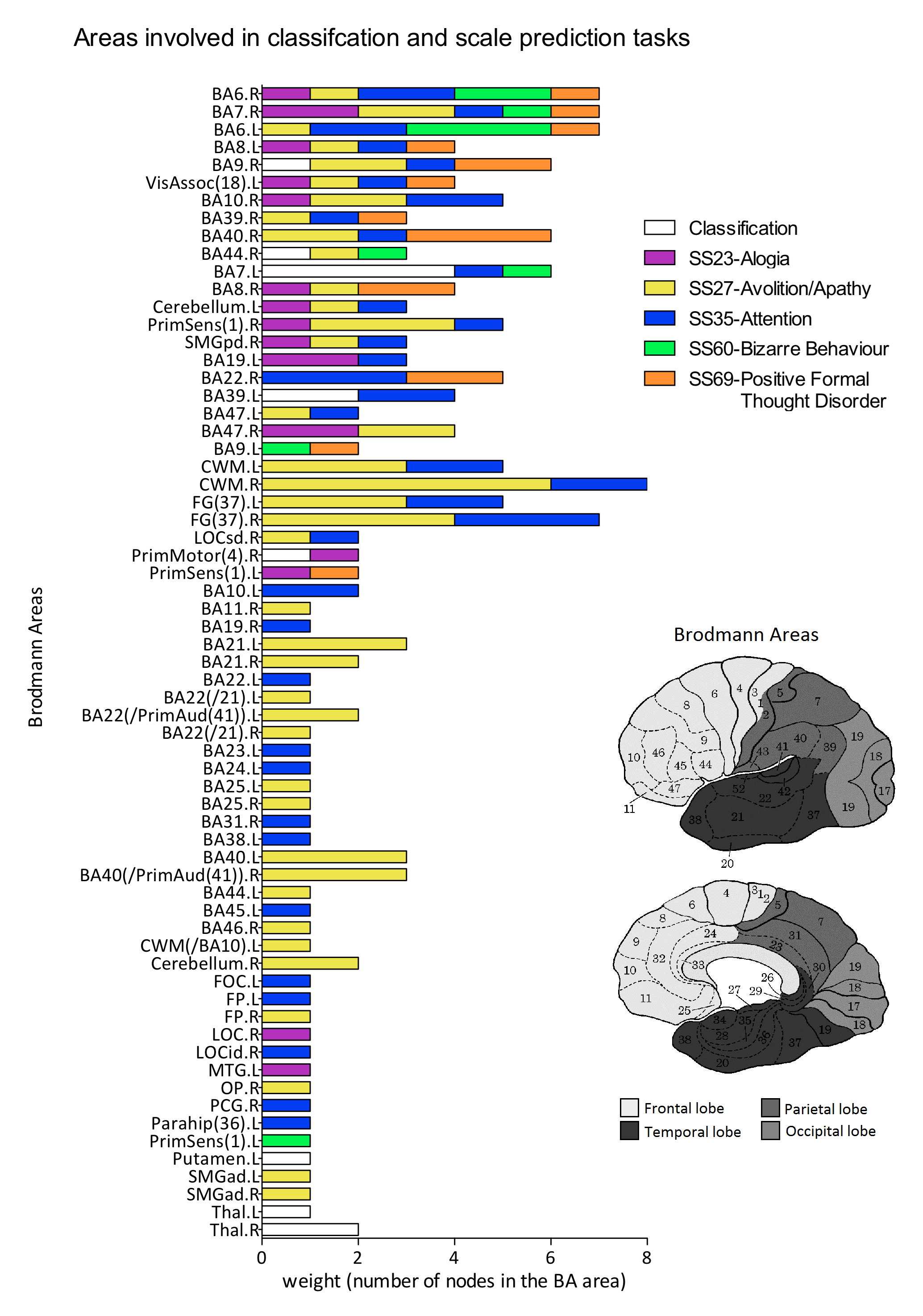
**

**Figure S9.** Summary of all areas corresponding to the stable most-predictive links involved in classification or scale prediction tasks, shown in different colors. The length of the bars shows the number of nodes in the corresponding area shown on the vertical axis. Areas are sorted based on the number of tasks they were involved in and then alphabetically for the areas that were involved only in one tasks. For instance, area BA6.R was involved in prediction of all five scales, or thalamus was only involved in classification. The brain picture with Brodmann’s parcellation is included as a reference to aid with the location of different Brodmann areas (adopted with permission from H. Elliott, Textbook of Neuroanatomy. Philadelphia: Lippincott^2^.)

**Supplementary Tables**

**Table S1**. Scanner and image acquisition information per site.

| Site code | MR Field strength | make | Head coil | k-space |
| --- | --- | --- | --- | --- |
| 3 | 1.5T, 4T | GE | Volume birdcage | (spiral) |
| 9 | 1.5T | Marconi | Bird-cage | linear |
| 10 | 1.5T | Siemens | CP for EPI; 8 channel for T1 | linear |
| 18 | 3T | Siemens | CP | linear |
| 6 | 3T | Siemens | 8-channel | linear |

**Table S2.** Motion parameters. Average of mean absolute and mean relative displacement motion parameters in healthy and patient group. The two mean displacement parameters for each subject are extracted from the output of the FSL MCFLIRT motion correction tool. Mean absolute displacement for each subject is the average displacement of volumes relative to the reference volume, and mean relative displacement is the average frame-to-frame displacement, which is computed separately for each run per subject.

|  | Median absolute disp. (mm) | Median relative disp. (mm) | Mean absolute disp. (S.D.) (mm) | Mean relative disp. (S.D.) (mm) |
| --- | --- | --- | --- | --- |
| Healthy controls | 0.1580 | 0.0566 | 0.2005 (0.1452) | 0.0742 (0.0632) |
| Patients | 0.1874 | 0.0827 | 0.2458 (0.1927) | 0.1066 (0.0755) |

**Table S3**. Demographic information of subjects in the study.

| Subject class | Age (S.D.) | Gender | | Handedness | | | IQ (S.D.)^ii^ | Medication^iii^ | |
| --- | --- | --- | --- | --- | --- | --- | --- | --- | --- |
|  |  | M | F | R | L | M^i^ |  | Yes | No |
| Healthy control | 36.57 (12.96) | 37 | 12 | 45 | 3 | 1 | 111.96 (8.84) | - | - |
| Patients | 38.98 (12.49) | 35 | 11 | 40 | 6 | - | 105.60 (7.70) | 39 | 3 |
| **Total** | **37.74 (12.72)** | **72** | **23** | **85** | **9** | **1** | **109.08 (8.89)** | **-** | **-** |

^i^M stands for mixed handed.

^ii^IQ denotes Full IQ score as measured by NAART. IQ scores were not available for seven patients and two controls.

^iii^Information about medication at the time of scans (yes/no) was not available for n = 4 out of 46 patients; In terms of medication history, only n = 1 out of three unmedicated patients was neuroleptic-naïve. Almost all other patients (n = 33), where data was available (n=34), were neuroleptic non-naïve.

**Table S4**. Subject combination per site. Baseline for classification based on majority class vote.

| Site code | Num Subjects | Num Scz | Num Ctrl | baseline for classification |
| --- | --- | --- | --- | --- |
| 3 | 21 | 10 | 11 | 0.5238 |
| 9 | 22 | 10 | 12 | 0.5455 |
| 10 | 23 | 10 | 13 | 0.5652 |
| 18 | 23 | 11 | 12 | 0.5217 |
| 6 | 6 | 5 | 1 | - |
| All sites | 95 | 46 | 49 | 0.5158 |
| **Total num samples** | **380** | **184** | **196** | **0.5158** |

**Table S5.** MNI X, Y, Z coordinates and broad anatomical and functional labels for the eight largest clusters (with > 10 voxels in the cluster) (identified by FSL cluster command) in the CV-stable statistical significance map for ss-log-degree feature shown in the figure below as well as Figure 1. (Note that the maps in Figure 1 are smoothed for visualization purposes.)

| Cluster Index | Voxels | MAX | MAX X (mm) | MAX Y (mm) | MAX Z (mm) | Gross Anatomical Label | Hemisphere | Brodmann Label |
| --- | --- | --- | --- | --- | --- | --- | --- | --- |
| 1 | 94 | 23 | -44.1 | 4.62 | 28 | Precentral Gyrus | L | BA6 |
| 2 | 47 | 24 | -30.3 | 4.62 | 48 | Middle Frontal Gyrus | L | BA6^*^ |
| 3 | 46 | 27 | -37.2 | -64.1 | 33 | Lateral Occipital Cortex, superior division | L | BA39 |
| 4 | 26 | 22 | -2.81 | -40.1 | 13 | Cingulate Gyrus, posterior division | L | BA30^*^ |
| 5 | 17 | 23 | -2.81 | -57.2 | 58 | Precuneous Cortex | L | BA7 |
| 6 | 15 | 21 | -2.81 | -43.5 | 43 | Precuneous Cortex | L | BA31 |
| 7 | 12 | 23 | -47.5 | -36.6 | 28 | Parietal Operculum Cortex | L | BA40^*^ |
| 8 | 11 | 27 | 55.6 | 8.06 | 28 | Precentral Gyrus | R | BA6 |

^*^Label of the closest region. Brodmann area labels are extracted from the BioImage Suite^3,4^ (see also Table S7 caption for more details on the structural atlases).

Figure: A stable subset of statistically significant (Bonferroni-surviving) site-standardized log-degree features, across 95 data subsets corresponding to leave-subject-out cross-validation, shown at the original fMRI resolution. The five largest clusters involving BA6, 39, 7, and 30 are noticeable in the figure. A smoothed version of this information is presented in Figure 1.

**Table S6.** Lowest cross-validation error rates achieved at k top-ranking number of variables for various classifiers using the ss-link-weights feature. (Error rate for all K values are shown in Figure 3; FP and FN rates are shown in the Supplementary Figure S4). Error of majority vote classifier is 0.4842.

| Feature: site standardized supervoxel-level link weights | | | | |
| --- | --- | --- | --- | --- |
| Classifier | Lowest error/  Highest accuracy | K | Next lowest error | Next best K |
| Nearest Neighbors | 0.276 (0.72) | 512 | 0.287 | 256 |
| Linear SVM | 0.268 (0.73) | 30 | 0.274 | 161,596 |
| Decision Tree | 0.353 (0.65) | 32 | 0.358 | 256 |
| Random Forest | 0.279 (0.72) | 30 | 0.289 | 512 |
| Logistic Regression | 0.260 (0.74) | 1024 | 0.279 | 32 |
| Naive Bayes | 0.279 (0.72) | 256 | 0.279 | 512 |
| LDA | 0.274 (0.73) | 16 | 0.282 | 32 |

**Table S7.** MNI coordinate and corresponding Brodmann areas (BA) and broad anatomies for the 15 nodes (supervoxels) involved in the top 13 CV-stable links illustrated in Figure 4. BioImage Suite online tool^3^ was used to convert MNI coordinates to Brodmann areas. MNI to Talairach mapping in the tool is based on Lacadie, et al. ^4^. Nodes broad anatomical label were also extracted using *fslatlasquery* tool and three atlases: Harvard_Oxford Cortical and/or Subcortical^5-8^, and MNI^9,10^ Structural atlases, covering 48, 21, and 9 structural areas, respectively. (For two out of 15 nodes, BIS did not associate with predefined BAs; for those, we considered the anatomical labels with the highest probability in the H.O. probabilistic atlases. Note that the labels provided correspond to the center of the supervoxels, a relatively large volume of size 13.75 x 13.75 x 15 mm (supervoxels do not overlap); It is possible that a supervoxel includes more than one structural region (for example node 4 center is associated with white matter, but it is also adjacent to subcortical areas Pallidum and Thalamus.) (BIS: BioImage Suite, H.O.: Harvard-Oxford, CWM: cerebral white matter, Thal: Thalamus)

| Node index | X | Y | Z | Brodmann label  (BIS) | Broad anatomical label  (H.O. cortical/subcortical atlases) | MNI structural  atlas |
| --- | --- | --- | --- | --- | --- | --- |
| 1 | 7.5 | -29.75 | 3 | Thal.R | Right Thal | Thal |
| 2 | 7.5 | -16 | 3 | Thal.R | Right Thal | Thal |
| 3 | -6.25 | -16 | 3 | Thal.L | Left Thal | Thal |
| 4 | -20 | -16 | 3 | - | Left CWM, (Left Pallidum, Left Thal) | Thal |
| 5 | 48.75 | 11.5 | 3 | BA44.R | Frontal Operculum Cortex, (Inferior Frontal Gyrus, pars opercularis, Precentral Gyrus) | Frontal Lobe |
| 6 | -20 | 11.5 | 3 | Putamen.L | Left Putamen | Putamen |
| 7 | -47.5 | -71 | 18 | BA39.L(nt19) | Lateral Occipital Cortex, superior division, (Lateral Occipital Cortex, inferior division) | Occipital Lobe |
| 8 | 35 | 39 | 18 | BA9.R | Right CWM, (Frontal Pole, Middle Frontal Gyrus) | Frontal Lobe |
| 9 | -61.25 | -43.5 | 33 | BA39.L | Supramarginal Gyrus, posterior division (Supramarginal Gyrus, anterior division, Angular Gyrus) | Parietal Lobe |
| 10 | -20 | -84.75 | 48 | - | Lateral Occipital Cortex, superior division | Occipital Lobe |
| 11 | -6.25 | -71 | 48 | BA7.L | Precuneous Cortex, (Lateral Occipital Cortex, superior division) | Parietal Lobe |
| 12 | -20 | -71 | 48 | BA7.L | Lateral Occipital Cortex, superior division, (Precuneous Cortex) | Parietal Lobe |
| 13 | 48.75 | -16 | 48 | PrimMotor(4).R | Postcentral Gyrus | Parietal Lobe (Frontal Lobe) |
| 14 | -6.25 | -57.25 | 63 | BA7.L | Precuneous Cortex | Parietal Lobe |
| 15 | -20 | -43.5 | 63 | BA7.L | Postcentral Gyrus, (Superior Parietal Lobule) | Parietal Lobe |

**Table S8.** MNI coordinates of the nodes involved in prediction of scales: (a) SS35; (b) SS60; (c) SS69; (d) SS27; and (e) SS23. (See Figure S7 caption for anatomical areas abbreviations, where the BIS^1^ tool did not provide BA labels.)

**Table S8a.**

| Scale: SS35 Attention | | |  |  |  |  |  |  |  |  |  |
| --- | --- | --- | --- | --- | --- | --- | --- | --- | --- | --- | --- |
| Node  index | X | Y | Z | Brodmann labels | Lobe | Node index | X | Y | Z | Brodmann labels | Lobe |
| 1 | -47.5 | -57.25 | -27 | Cerebellum.L | C | 25 | -33.75 | -43.5 | 18 | CWM.L | - |
| 2 | 48.75 | -29.75 | -27 | FG(37).R | T | 26 | -61.25 | -43.5 | 18 | BA39.L | P |
| 3 | -33.75 | -16 | -27 | Parahip(36).L | T | 27 | 21.25 | -29.75 | 18 | CWM.R | - |
| 4 | -20 | 11.5 | -27 | FOC.L | F | 28 | 62.5 | 11.5 | 18 | PCG.R | F |
| 5 | -47.5 | 11.5 | -27 | BA38.L | T | 29 | -33.75 | 25.25 | 18 | CWM.L | - |
| 6 | 35 | -71 | -12 | BA19.R | O | 30 | 21.25 | 52.75 | 18 | BA10.R | F |
| 7 | 48.75 | -57.25 | -12 | FG(37).R | T | 31 | -20 | 66.5 | 18 | FP | F |
| 8 | -20 | -43.5 | -12 | FG(37).L | O | 32 | 35 | -84.75 | 33 | BA39.R | P |
| 9 | -47.5 | -43.5 | -12 | - | T | 33 | -33.75 | -84.75 | 33 | BA19.L | O |
| 10 | 62.5 | -2.25 | -12 | BA22.R | T | 34 | -33.75 | -71 | 33 | BA39.L | P |
| 11 | 48.75 | 25.25 | -12 | BA22.R | F | 35 | 7.5 | -57.25 | 33 | BA31.R | P |
| 12 | -33.75 | 25.25 | -12 | BA47.L | F | 36 | 48.75 | -29.75 | 33 | BA40.R | P |
| 13 | -6.25 | 52.75 | -12 | BA10.L | F | 37 | 48.75 | -16 | 33 | PrimSens(1).R | P |
| 14 | 48.75 | -84.75 | 3 | LOCad.R | O | 38 | 21.25 | 11.5 | 33 | CWM.R | - |
| 15 | 62.5 | -57.25 | 3 | FG(37).R | T | 39 | 21.25 | 52.75 | 33 | BA9.R | F |
| 16 | -47.5 | -57.25 | 3 | FG(37).L | O | 40 | -6.25 | -71 | 48 | BA7.L | P |
| 17 | -61.25 | -29.75 | 3 | BA22.L | T | 41 | 21.25 | -57.25 | 48 | LOCsd.R | P |
| 18 | 62.5 | -2.25 | 3 | BA22.R | T | 42 | 7.5 | -57.25 | 48 | BA7.R | P |
| 19 | -6.25 | 39 | 3 | BA24.L | F | 43 | 62.5 | -43.5 | 48 | SMGpd.R | P |
| 20 | -47.5 | 39 | 3 | BA45.L | F | 44 | -6.25 | -2.25 | 48 | BA6.L | F |
| 21 | 48.75 | 52.75 | 3 | BA10.R | F | 45 | -20 | 11.5 | 48 | BA6.L | F |
| 22 | -6.25 | 66.5 | 3 | BA10.L | F | 46 | -20 | 25.25 | 48 | BA8.L | F |
| 23 | -6.25 | -98.5 | 18 | VisAssoc(18).L | O | 47 | 35 | -16 | 63 | BA6.R | F |
| 24 | -6.25 | -43.5 | 18 | BA23.L | P | 48 | 21.25 | 11.5 | 63 | BA6.R | F |
| T: Temporal; F: Frontal; P: Parietal; O: Occipital; C: Cerebellum; CWM: Cerebral White Matter | | | | | | | | |  |  |  |

**Table S8b.**

| Scale: SS60 | Bizarre behaviour | |  |  |
| --- | --- | --- | --- | --- |
| Node index | X | Y | Z | Brodmann labels |
| 1 | 48.75 | 11.5 | 33 | BA44.R |
| 2 | -20 | 39 | 33 | BA9.L |
| 3 | 7.5 | -57.25 | 48 | BA7.R |
| 4 | -6.25 | -57.25 | 48 | BA7.L |
| 5 | 62.5 | -29.75 | 48 | SMGad.R |
| 6 | -6.25 | -16 | 48 | BA6.L |
| 7 | -6.25 | -2.25 | 48 | BA6.L |
| 8 | 7.5 | 11.5 | 48 | BA6.R |
| 9 | -6.25 | 11.5 | 48 | BA6.L |
| 10 | -20 | -29.75 | 63 | PrimSens(1).L |
| 11 | 7.5 | -2.25 | 63 | BA6.R |

**Table S8c.**

| Scale: SS69 | Positive formal thought disorder | | | |
| --- | --- | --- | --- | --- |
| Node index | X | Y | Z | Brodmann labels |
| 1 | 62.5 | -29.75 | 3 | BA22.R |
| 2 | 62.5 | -2.25 | 3 | BA22.R |
| 3 | -6.25 | -98.5 | 18 | VisAssoc(18).L |
| 4 | 35 | 39 | 18 | BA9.R |
| 5 | 62.5 | -43.5 | 33 | BA40.R |
| 6 | 48.75 | -43.5 | 33 | BA40.R |
| 7 | 48.75 | -29.75 | 33 | BA40.R |
| 8 | 35 | 39 | 33 | BA9.R |
| 9 | -33.75 | 39 | 33 | BA9.L |
| 10 | 35 | -71 | 48 | BA39.R |
| 11 | 7.5 | -71 | 48 | BA7.R |
| 12 | 7.5 | 25.25 | 48 | BA8.R |
| 13 | -6.25 | 25.25 | 48 | BA8.L |
| 14 | 7.5 | 39 | 48 | BA8.R |
| 15 | -6.25 | -29.75 | 63 | PrimSens(1).L |
| 16 | 7.5 | 25.25 | 63 | BA6.R |
| 17 | -6.25 | 25.25 | 63 | BA6.L |

**Table S8d.**

| Scale: SS27 | Avolition-Apathy | |  |  |  |  |  |  |  |
| --- | --- | --- | --- | --- | --- | --- | --- | --- | --- |
| Node index | X | Y | Z | Brodmann labels | Node index | X | Y | Z | Brodmann labels |
| 1 | 48.75 | -71 | -27 | Cerebellum.R | 33 | -20 | -29.75 | 18 | CWM.L |
| 2 | -47.5 | -71 | -27 | Cerebellum.L | 34 | 48.75 | -16 | 18 | PrimSens(1).R |
| 3 | 48.75 | -57.25 | -27 | Cerebellum.R | 35 | -20 | -16 | 18 | CWM.L |
| 4 | 35 | -29.75 | -27 | FG(37).R | 36 | 48.75 | 25.25 | 18 | BA9.R |
| 5 | 48.75 | -71 | -12 | FG(37).R | 37 | -47.5 | 25.25 | 18 | BA44.L |
| 6 | 48.75 | -57.25 | -12 | FG(37).R | 38 | 48.75 | 39 | 18 | BA46.R |
| 7 | -61.25 | -57.25 | -12 | FG(37).L | 39 | 35 | 39 | 18 | BA9.R |
| 8 | -61.25 | -43.5 | -12 | FG(37).L | 40 | 21.25 | 39 | 18 | CWM.R |
| 9 | 62.5 | -29.75 | -12 | BA21.R | 41 | 21.25 | 66.5 | 18 | BA10.R |
| 10 | -61.25 | -29.75 | -12 | BA21.L | 42 | 21.25 | -57.25 | 33 | CWM.R |
| 11 | 62.5 | -16 | -12 | BA21.R | 43 | 62.5 | -43.5 | 33 | BA40.R |
| 12 | -61.25 | -16 | -12 | BA21.L | 44 | 48.75 | -43.5 | 33 | BA40.R |
| 13 | 7.5 | 11.5 | -12 | BA25.R | 45 | -33.75 | -43.5 | 33 | CWM.L |
| 14 | -6.25 | 11.5 | -12 | BA25.L | 46 | -47.5 | -29.75 | 33 | BA40.L |
| 15 | 48.75 | 25.25 | -12 | BA47.R | 47 | -61.25 | -29.75 | 33 | BA40.L |
| 16 | 35 | 39 | -12 | BA47.R | 48 | -61.25 | -2.25 | 33 | BA6.L |
| 17 | 21.25 | 39 | -12 | BA11.R | 49 | 48.75 | 11.5 | 33 | BA44.R |
| 18 | -47.5 | 39 | -12 | BA47.L | 50 | 21.25 | -84.75 | 48 | LOCsd.R |
| 19 | 48.75 | -57.25 | 3 | FG(37).R | 51 | 35 | -71 | 48 | BA39.R |
| 20 | -47.5 | -57.25 | 3 | FG(37).L | 52 | 62.5 | -43.5 | 48 | SMGpd.R |
| 21 | -47.5 | -43.5 | 3 | BA21.L | 53 | 35 | -43.5 | 48 | BA7.R |
| 22 | 48.75 | -29.75 | 3 | BA22.R/21) | 54 | 21.25 | -43.5 | 48 | CWM.R |
| 23 | -61.25 | -29.75 | 3 | BA22.L/21) | 55 | 62.5 | -29.75 | 48 | SMGad.R |
| 24 | -61.25 | -16 | 3 | BA22.L/PrimAud(41) | 56 | -47.5 | -29.75 | 48 | BA40.L |
| 25 | 35 | 39 | 3 | CWM.R | 57 | -61.25 | -29.75 | 48 | SMGad.L |
| 26 | 21.25 | 39 | 3 | CWM.R | 58 | 35 | 25.25 | 48 | BA8.R |
| 27 | 35 | 52.75 | 3 | BA10.R | 59 | -33.75 | 25.25 | 48 | BA8.L |
| 28 | -20 | 52.75 | 3 | CWM/BA10 | 60 | 7.5 | 52.75 | 48 | FP.R |
| 29 | 7.5 | -98.5 | 18 | OP.R | 61 | 21.25 | -57.25 | 63 | BA7.R |
| 30 | -6.25 | -98.5 | 18 | VisAssoc(18).L | 62 | 48.75 | -29.75 | 63 | PrimSens(1).R |
| 31 | 35 | -29.75 | 18 | BA40.R/PrimAud(41) | 63 | 35 | -29.75 | 63 | PrimSens(1).R |
| 32 | 21.25 | -29.75 | 18 | CWM.R | 64 | 7.5 | 25.25 | 63 | BA6.R |

**Table S8e.**

| Scale: SS23 | Alogia | |  |  |
| --- | --- | --- | --- | --- |
| Node index | X | Y | Z | Brodmann labels |
| 1 | -20 | -84.75 | -27 | Cerebellum.L |
| 2 | -20 | -71 | -12 | VisAssoc(18).L |
| 3 | -20 | -57.25 | -12 | BA19.L |
| 4 | -61.25 | -2.25 | -12 | MTG.L |
| 5 | 48.75 | 25.25 | -12 | BA47.R |
| 6 | 48.75 | 39 | -12 | BA47.R |
| 7 | 21.25 | 52.75 | -12 | BA10.R |
| 8 | -33.75 | -84.75 | 33 | BA19.L |
| 9 | 21.25 | -84.75 | 48 | LOC.R |
| 10 | 7.5 | -71 | 48 | BA7.R |
| 11 | 62.5 | -43.5 | 48 | SMGpd.R |
| 12 | 48.75 | -16 | 48 | PrimMot(4).R |
| 13 | -47.5 | -16 | 48 | PrimSens(1).L |
| 14 | 7.5 | 25.25 | 48 | BA8.R |
| 15 | -6.25 | 25.25 | 48 | BA8.L |
| 16 | 21.25 | -71 | 63 | BA7.R |
| 17 | 35 | -29.75 | 63 | PrimSens(1).R |
| 18 | 7.5 | 25.25 | 63 | BA6.R |

**Table S9.** Summary of the stable predictive links involved in classification. (Links are displayed in Figure 4; see Table S7 for the MNI coordinates of all nodes in the following table.)

| Node_index | From | <-> | To | Strength |
| --- | --- | --- | --- | --- |
| 1 | PrimMotor(4).R | <-> | Thal.R | 40.58 |
| 2 | BA7.L | <-> | Thal.R | 42.52 |
| 3 | LOCsd.L | <-> | Thal.R | 40.86 |
| 4 | PrimMotor(4).R | <-> | Thal.R | 43.98 |
| 5 | BA7.L | <-> | Thal.R | 39.87 |
| 6 | LOCsd.L | <-> | Thal.L | 39.44 |
| 7 | BA7.L | <-> | Thal.L | 39.24 |
| 8 | LOCsd.L | <-> | CWM(ntPutamen).L^*^ | 42.2 |
| 9 | BA7.L | <-> | CWM(ntPutamen).L | 42.24 |
| 10 | BA7.L | <-> | BA44.R | 38.92 |
| 11 | BA7.L | <-> | Putamen.L | 39.17 |
| 12 | BA39.L | <-> | BA39(nt19).L | 41.48 |
| 13 | BA7.L | <-> | BA9.R | 40.06 |

^*^nt: next to

Same as before, but the links are sorted based on their weights.

| Link index |  |  |  | Link strength |
| --- | --- | --- | --- | --- |
| 1 | Thal.R | <-> | PrimMotor(4).R | 43.98 |
| 2 | Thal.R | <-> | BA7.L | 42.52 |
| 3 | CWM(ntPutamen).L | <-> | BA7.L | 42.24 |
| 4 | CWM(ntPutamen).L | <-> | LOCsd.L | 42.2 |
| 5 | BA39(nt19).L | <-> | BA39.L | 41.48 |
| 6 | Thal.R | <-> | LOCsd.L | 40.86 |
| 7 | Thal.R | <-> | PrimMotor(4).R | 40.58 |
| 8 | BA9.R | <-> | BA7.L | 40.06 |
| 9 | Thal.R | <-> | BA7.L | 39.87 |
| 10 | Thal.L | <-> | LOCsd.L | 39.44 |
| 11 | Thal.L | <-> | BA7.L | 39.24 |
| 12 | Putamen.L | <-> | BA7.L | 39.17 |
| 13 | BA44.R | <-> | BA7.L | 38.92 |

**Table S10.** Summary of stable links involved in prediction of symptom scales: (a) SS35; (b) SS60; (c) SS69; (d) SS27; and (e) SS23. (The order of scales is based on the value of their Spearman correlation coefficient in Table 1.) (See Figure S7a-e for links displayed on brain templates; see Table S8 for each node’s MNI coordinate; See Figure S7 caption for areas abbreviations.) The link strength in each row represents the average coefficient of the link in the best EN model discussed in section 3.4 and shown in Supplementary Figure S7.

**Table S10a.**

| SS35 | Attention |  |  |  |
| --- | --- | --- | --- | --- |
| Link_index | From | <-> | To | Strength |
| 1 | BA19.R | <-> | Cerebellum.L | 0.73 |
| 2 | LOCsd.R | <-> | FG(37).R | 0.37 |
| 3 | BA22.R | <-> | Parahip(36).L | 0.33 |
| 4 | PCG.R | <-> | Parahip(36).L | 0.4 |
| 5 | CWM.L | <-> | Parahip(36).L | 0.26 |
| 6 | CWM.R | <-> | Parahip(36).L | 0.23 |
| 7 | BA7.L | <-> | Parahip(36).L | 0.28 |
| 8 | CWM.L | <-> | FOC.L | 0.19 |
| 9 | BA40.R | <-> | BA38.L | 0.44 |
| 10 | BA39.R | <-> | FG(37).R | 0.49 |
| 11 | BA19.L | <-> | FG(37).R | 0.42 |
| 12 | BA39.R | <-> | FG(37).L | 0.37 |
| 13 | FP.L | <-> | - | 0.51 |
| 14 | LOCid.R | <-> | BA22.R | 0.35 |
| 15 | BA6.L | <-> | BA22.R | 0.86 |
| 16 | FG(37).L | <-> | BA47.L | 0.25 |
| 17 | PrimSens(1).R | <-> | BA10.L | 0.26 |
| 18 | BA22.L | <-> | FG(37).R | 0.42 |
| 19 | BA24.L | <-> | BA22.R | 0.47 |
| 20 | BA6.L | <-> | BA45.L | 0.53 |
| 21 | BA6.R | <-> | BA10.R | 0.61 |
| 22 | FP.L | <-> | BA10.L | 0.51 |
| 23 | BA7.R | <-> | VisAssoc(18).L | 0.52 |
| 24 | CWM.R | <-> | BA23.L | 0.27 |
| 25 | BA8.L | <-> | BA39.L | 0.36 |
| 26 | BA39.L | <-> | BA10.R | 0.56 |
| 27 | BA9.R | <-> | BA31.R | 0.32 |
| 28 | BA6.R | <-> | SMGpd.R | 0.72 |

**Table S10b.**

| Scale: SS60 | Bizarre behaviour |  |  |  |
| --- | --- | --- | --- | --- |
| Link_index | From | <-> | To | Strength |
| 1 | BA6.L | <-> | BA44.R | 0.39 |
| 2 | PrimSens(1).L | <-> | BA44.R | 0.68 |
| 3 | - | <-> | BA9.L | 0.86 |
| 4 | BA6.R | <-> | BA7.R | 0.85 |
| 5 | BA6.L | <-> | BA7.R | 0.4 |
| 6 | BA6.R | <-> | BA7.R | 0.9 |
| 7 | BA6.L | <-> | BA7.L | 0.46 |
| 8 | BA6.R | <-> | BA7.L | 1.22 |
| 9 | BA6.R | <-> | BA7.L | 0.5 |

**Table S10c.**

| Scale: SS69 | Positive formal thought disorder | | |  |
| --- | --- | --- | --- | --- |
| Link_index | From | <-> | To | Strength |
| 1 | BA40.R | <-> | BA22.R | 0.88 |
| 2 | BA9.R | <-> | BA22.R | 0.34 |
| 3 | BA39.R | <-> | VisAssoc(18).L | 1.07 |
| 4 | BA40.R | <-> | BA40.R | 0.3 |
| 5 | BA6.L | <-> | BA9.R | 0.55 |
| 6 | BA8.R | <-> | BA9.L | 1.07 |
| 7 | BA8.L | <-> | BA9.L | 1.64 |
| 8 | BA8.R | <-> | BA9.L | 0.6 |
| 9 | BA6.R | <-> | BA9.L | 1.39 |
| 10 | BA6.L | <-> | BA9.L | 1.23 |
| 11 | PrimSens(1).L | <-> | BA7.R | 0.98 |

**Table S10d.**

| Scale: SS27 | Avolition-Apathy |  |  |  |
| --- | --- | --- | --- | --- |
| Link_index | From | <-> | To | Strength |
| 1 | BA40.L | <-> | Cerebellum.R | 0.38 |
| 2 | BA10.R | <-> | Cerebellum.L | 0.41 |
| 3 | BA39.R | <-> | Cerebellum.R | 0.67 |
| 4 | FG(37).R | <-> | FG(37).R | 0.86 |
| 5 | BA11.R | <-> | FG(37).R | 0.53 |
| 6 | CWM.R | <-> | FG(37).L | 0.41 |
| 7 | BA47.R | <-> | FG(37).L | 0.52 |
| 8 | BA21.L | <-> | BA21.R | 0.57 |
| 9 | BA25.R | <-> | BA21.L | 0.43 |
| 10 | BA25.L | <-> | BA21.L | 0.85 |
| 11 | BA22(/21).L | <-> | BA21.R | 0.97 |
| 12 | BA22(/PrimAud(41)).L | <-> | BA21.R | 1.25 |
| 13 | FG(37).L | <-> | BA21.L | 0.62 |
| 14 | BA21.L | <-> | BA21.L | 1.1 |
| 15 | CWM.L/BA10 | <-> | BA21.L | 0.77 |
| 16 | BA9.R | <-> | BA47.R | 0.64 |
| 17 | BA44.R | <-> | BA47.R | 0.76 |
| 18 | BA44.L | <-> | BA47.L | 0.49 |
| 19 | BA6.R | <-> | FG(37).R | 0.37 |
| 20 | BA6.L | <-> | BA22(/21).R | 0.51 |
| 21 | BA7.R | <-> | CWM.R | 0.53 |
| 22 | BA7.R | <-> | CWM.R | 0.78 |
| 23 | FP.R | <-> | OP.R | 0.46 |
| 24 | BA46.R | <-> | VisAssoc(18).L | 0.79 |
| 25 | CWM.R | <-> | BA40(/PrimAud(41)).R | 0.52 |
| 26 | CWM.L | <-> | BA40(/PrimAud(41)).R | 0.69 |
| 27 | PrimSens(1).R | <-> | CWM.R | 0.59 |
| 28 | PrimSens(1).R | <-> | CWM.R | 0.28 |
| 29 | PrimSens(1).R | <-> | CWM.R | 0.51 |
| 30 | BA40.L | <-> | CWM.L | 0.51 |
| 31 | BA7.R | <-> | BA9.R | 0.54 |
| 32 | BA8.R | <-> | BA10.R | 0.71 |
| 33 | BA8.L | <-> | BA10.R | 1.08 |
| 34 | CWM.L | <-> | CWM.R | 0.78 |
| 35 | BA40.L | <-> | BA40.R | 0.93 |
| 36 | BA40.L | <-> | BA40.R | 0.48 |
| 37 | LOCsd.R | <-> | BA40.L | 0.88 |
| 38 | SMGpd | <-> | BA40.L | 1.13 |
| 39 | SMGad.R | <-> | BA40.L | 0.52 |
| 40 | SMGad.L | <-> | LOCsd.R | 0.64 |
| 41 | BA7.R | <-> | BA7.R | 0.71 |
| 42 | BA7.R | <-> | CWM.R | 0.61 |

**Table S10e.**

| Scale: SS23 | Alogia |  |  |  |
| --- | --- | --- | --- | --- |
| Link_index | From | <-> | To | Strength |
| 1 | PrimSens(1).L | <-> | Cerebellum.L | 1.33 |
| 2 | BA6.R | <-> | VisAssoc(18).L | 0.46 |
| 3 | BA8.R | <-> | BA19.L | 0.71 |
| 4 | BA8.L | <-> | BA19.L | 0.75 |
| 5 | PrimMotor(4).R | <-> | MTG.L | 0.46 |
| 6 | PrimSens(1).R | <-> | MTG.L | 0.76 |
| 7 | BA19.L | <-> | BA47.R | 0.82 |
| 8 | BA7.R | <-> | BA47.R | 0.48 |
| 9 | BA19.L | <-> | BA10.R | 0.55 |
| 10 | SMG.R | <-> | LOC.R | 0.59 |
| 11 | BA7.R | <-> | SMG.R | 1.31 |

**Appendix**

**Figure A1.** Predictive accuracy of the Elastic Net model for nine Global Rating SANS and SAPS scales, measured by Spearman Correlation between the actual and predicted scale values, for a range of sparsity parameter (x-axis, number of variables selected) and grouping parameter λ_2_.

**Figure A2.** Stability of features selected by the *best* EN model for each of the FDR surviving scales, shown as the number of features (y-axis) selected *k* times (x-axis), where *k* = {1, 2, 3, …, CV-folds}, across the leave-one-subject-out data subsets. The CV stable link maps in figure S7, illustrate the features that are selected in all CV folds (*N* = 46 for SS23, SS27, SS35, SS60, and *N* = 45 for SS69).

**Figure A3.** Coefficients of features (supervoxel-level link-weights) in the *best* EN model averaged over CV folds, ranked from left to right based on the number of times they were selected. Different panels show the results for the 5 FDR surviving scales. The x-axis shows the features indices starting from 1 up to the total number of the features selected across all CV fold data subsets (i.e. the union of the features selected across CV folds). The vertical dotted lines separate the features based on the number of times they were selected. For example, in the last scale (SS69), 11 data points are in the first parcellation (*k* = 45), meaning that the 11 corresponding features were selected in all CV folds. The last parcellation, where most data points fall into (*k* = 1), shows the features that were selected only once. (Note that the average is taken over nonzero coefficients only, thus for features that were selected only once (*k* = 1), the average coefficient equaled the feature coefficient in only one model.) In general, the coefficients were larger for the features that were selected in all folds (i.e. the left most parcels).

**References for Supplementary Material:**

1 Behzadi, Y., Restom, K., Liau, J. & Liu, T. T. A component based noise correction method (CompCor) for BOLD and perfusion based fMRI. *Neuroimage* **37**, 90-101, doi:10.1016/j.neuroimage.2007.04.042 (2007).

2 Elliott, H. *Textbook of Neuroanatomy*. 2nd edn, (J. B. Lippincott, 1969).

3 Papademetris, X. *et al.* BioImage Suite: An integrated medical image analysis suite: An update. *Insight J* **2006**, 209 (2006).

4 Lacadie, C. M., Fulbright, R. K., Rajeevan, N., Constable, R. T. & Papademetris, X. More accurate Talairach coordinates for neuroimaging using non-linear registration. *Neuroimage* **42**, 717-725, doi:10.1016/j.neuroimage.2008.04.240 (2008).

5 Makris, N. *et al.* Decreased volume of left and total anterior insular lobule in schizophrenia. *Schizophr Res* **83**, 155-171, doi:10.1016/j.schres.2005.11.020 (2006).

6 Frazier, J. A. *et al.* Structural brain magnetic resonance imaging of limbic and thalamic volumes in pediatric bipolar disorder. *Am J Psychiatry* **162**, 1256-1265, doi:10.1176/appi.ajp.162.7.1256 (2005).

7 Desikan, R. S. *et al.* An automated labeling system for subdividing the human cerebral cortex on MRI scans into gyral based regions of interest. *Neuroimage* **31**, 968-980, doi:10.1016/j.neuroimage.2006.01.021 (2006).

8 Goldstein, J. M. *et al.* Hypothalamic abnormalities in schizophrenia: sex effects and genetic vulnerability. *Biol Psychiatry* **61**, 935-945, doi:10.1016/j.biopsych.2006.06.027 (2007).

9 Collins, D. L., Holmes, C. J., Peters, T. M. & Evans, A. C. Automatic 3-D model-based neuroanatomical segmentation. *Hum Brain Mapp* **3**, 190-208 (1995).

10 Mazziotta, J. *et al.* A probabilistic atlas and reference system for the human brain: International Consortium for Brain Mapping (ICBM). *Philos Trans R Soc Lond B Biol Sci* **356**, 1293-1322, doi:10.1098/rstb.2001.0915 (2001).
